# MicroRNA-143 acts as a tumor suppressor through Musashi-2/DLL1/Notch1 and Musashi-2/Snail1/MMPs axes in acute myeloid leukemia

**DOI:** 10.1186/s12967-023-04106-6

**Published:** 2023-05-06

**Authors:** Fanfan Li, Yixiang Han, Rongrong Chen, Yinyan Jiang, Cheng Chen, Xiaofang Wang, Jifan Zhou, Qingqing Xu, Songfu Jiang, Si Zhang, Kang Yu, Shenghui Zhang

**Affiliations:** 1 Department of Hematology, The First Affiliated Hospital of Wenzhou Medical University, Wenzhou, 325015 Zhejiang China; 2 Institute of Hematology, Wenzhou Medical University, Wenzhou, Zhejiang China; 3 Wenzhou Key Laboratory of Hematology, Wenzhou, 325015 Zhejiang China; 4 Central Laboratory, The First Affiliated Hospital of Wenzhou Medical University, Wenzhou, Zhejiang China; 5 Department of Pathology, The First Affiliated Hospital of Wenzhou Medical University, Wenzhou, Zhejiang China; 6 Department of Biochemistry and Molecular Biology, NHC Key Laboratory of Glycoconjugates Research, School of Basic Medical Sciences, Fudan University, Shanghai, China; 7 Laboratory Animal Center, The First Affiliated Hospital of Wenzhou Medical University, Wenzhou, Zhejiang China

**Keywords:** Acute myeloid leukemia, microRNA-143, Musashi-2, DLL1, Snail1

## Abstract

**Background:**

The previous studies have revealed that abnormal RNA-binding protein Musashi-2 (MSI2) expression is associated with cancer progression through post-transcriptional mechanisms, however mechanistic details of this regulation in acute myeloid leukemia (AML) still remain unclear. Our study aimed to explore the relationship between microRNA-143 (miR-143) and MSI2 and to clarify their clinical significance, biological function and mechanism.

**Methods:**

Abnormal expression of miR-143 and MSI2 were evaluated in bone marrow samples from AML patients by quantitative real time-PCR. Effects of miR-143 on regulating MSI2 expression were investigated using luciferase reporter assay. Functional roles of MSI2 and miR-143 on AML cell proliferation and migration were determined by CCK-8 assay, colony formation, and transwell assays in vitro and in mouse subcutaneous xenograft and orthotopic transplantation models in vivo. RNA immunoprecipitation, RNA stability measurement and Western blotting were performed to assess the effects of MSI2 on AML.

**Results:**

We found that MSI2 was significantly overexpressed in AML and exerted its role of promoting AML cell growth by targeting DLL1 and thereby activating Notch signaling pathway. Moreover, we found that MSI2 bound to Snail1 transcript and inhibited its degradation, which in turn upregulated the expression of matrix metalloproteinases. We also found that MSI2 targeting miR-143 is downregulated in AML. In the AML xenograft mouse model, overexpression of MSI2 recapitulated its leukemia-promoting effects, and overexpression of miR-143 partially attenuated tumor growth and prevented metastasis. Notably, low expression of miR-143, and high expression of MSI2 were associated with poor prognosis in AML patients.

**Conclusions:**

Our data demonstrate that MSI2 exerts its malignant properties via DLL1/Notch1 cascade and the Snail1/MMPs axes in AML, and upregulation of miR-143 may be a potential therapeutic approach for AML.

**Supplementary Information:**

The online version contains supplementary material available at 10.1186/s12967-023-04106-6.

## Background

Acute myeloid leukemia (AML) is a heterogenous hematological malignancy characterized by inhibited differentiation and uncontrolled proliferation of hematopoietic stem/progenitor cells (HSPC). It is the most common leukemia in adults with the 5 year relative survival rate up to 29.5% [1]. While the genetic and epigenetic mechanisms of AML are being studied in depth, it is not yet mechanistically defined how post-transcriptional regulation of messenger RNA (mRNA) contributes to leukemia progression [2, 3].

*Musashi* (*Msi*) gene family encodes a group of RNA-binding proteins (RBPs) that post‐transcriptionally regulates mRNA processing by binding to recognition motifs located in the 3’ untranslated regions (UTRs) of target mRNAs [3, 4]. In mammals, two homologues of the MSI protein, MSI1 and MSI2, are highly expressed in stem cell compartments and aggressive tumors, including hematological malignancies [5–8], as they are emerging as regulators for mRNA stability and translation of proteins involved in essential oncogenic signaling pathways [9–13]. Accumulating data have confirmed that high expression of *Msi2* mRNA is associated with poor prognosis in AML, as patients with as low as 1% of total bone marrow cells expressing elevated MSI2 levels still have a short survival [14, 15]. Therefore MSI2 can be regarded as a hematopoietic oncogene with prognostic value.

Extramedullary AML sometimes involves the central nervous system and other solid organs, which is commonly associated with the former French-American-British (FAB) subtypes of AML-M5 [16]. Accordingly, increasing evidence supports the involvement of epithelial-mesenchymal transition (EMT) in hematologic diseases, enabling cancer cells to acquire migratory, invasive, and stem-like properties [17–20]. It is well known that one of the major triggering events of EMT is the activation of EMT-transcription factors, such as Snail1, an important transcription factor involved in EMT and invasion by decreasing the expression of E-cadherin and increasing matrix metalloproteinase (MMPs) [18, 21]. Although multiple RNA binding proteins and microRNAs (miRNAs) have been reported to promote EMT progression in solid tumors [22, 23], the roles and the underlying mechanisms of these post-transcriptional regulators have not been intensively investigated in mediating extramedullary metastasis of AML.

The miRNAs are a class of endogenous small non-coding RNAs that regulate their cognate mRNA transcripts at the translation level, in consequence playing important roles in cellular signaling cascades [24]. MiR-143 has been studied extensively for its role in neoplastic pathways in epithelial cell malignancies [25–27]. However, the role of miR-143 in hematological tumors has rarely been characterized.

Here, our mechanistic studies revealed that MSI2 activated the Notch signaling pathway and metastasis-related pathway through post-transcriptional upregulation of DLL1 and Snail1, thereby promoting AML cell proliferation and migration in vitro and accelerating AML progression in vivo. Further experiments validated that miR-143 could directly bind to MSI2 and enforced expression of miR-143 via intratumoral delivery had been shown to reduce the growth of AML xenografts in vivo.

## Methods

### Cell lines and primary AML cells

Human AML cell lines HL-60, HEL, THP-1 and KG-1α were acquired from ATCC. Primary AML cells were obtained from bone marrow (BM) aspirates of 58 newly diagnosed AML patients and the leukemia blasts were enriched by CD34 Diamond Microbeads (Miltenyi Biotec, Bergisch Gladbach, Germany). BM aspirates from 14 healthy donors served as negative control. All of these participants were from the First Affiliated Hospital of Wenzhou Medical University between January 1, 2019 and December 31, 2021 (Additional file 2: Table S1). Participant consent and approval were obtained from the Institutional Ethics Review Committee of the First Affiliated Hospital of Wenzhou Medical University for the use of human samples.

### Quantitative real-time PCR for gene expression assay

AML cells were transfected with siRNAs or miRNA mimic (GenePharma, Shanghai, CN) using Lipofectamine^®^ RNAiMAX Reagent (Invitrogen, CA, USA) and then identified by quantitative polymerase chain reaction (qPCR) analysis. The sequences of siRNAs and miRNAs were shown in Additional file 1: Table S2. Total RNA was extracted from AML cells using TRIzol (Sigma, MO, USA) and further transcribed into cDNA using PrimeScript^™^ RT Master Mix (Perfect Real Time) (Takara, Japan). Then, the cDNA was amplified using TB Green^®^ *Premix Ex Taq* II (Takara) on a 7500 Real-Time PCR System (Applied Biosystems, Foster City, CA, USA). MiR-143 was determined using Hairpin-it^™^ microRNA and U6 snRNA Normalization RT-PCR Quantitation Kit (GenePharma). The mRNA levels of protein-coding genes were normalized to GAPDH endogenous control and calculated according to the Pfaffl method [28]. Primers (Sangon Biotech, Shanghai, CN) were shown in Additional file 1: Table S3.

### Cell viability and colony formation assay

Cells transfected with siRNAs or miRNA mimic were seeded at a density of 5 × 10^3^/well in 96-well plates for 0, 24, 48, 72, 96 h. Cell viability was detected by Cell Counting Kit-8 (CCK-8; MedChemExpress, Shanghai, CN) assay and the absorbance at 450 nm was read on a Synergy H1 microplate reader (BioTek, Shoreline, WA, USA). After transfection as described above for 24 h, cells were plated into complete methylcellulose-based medium containing hIL-3, hGM-CSF and hSCF (MethoCult^™^ H4534 Classic Without EPO; STEMCELL Technologies, Cambridge, MA, USA) to assess their clonogenic capacity. Localized clusters of ≥ 50 cells were counted as colonies after 7–14 d. Then, the wells were washed twice with PBS and cells were counted manually.

### Cell migration and invasion assays

Transfected AML cells (1 × 10^5^ cells/well) were suspended in 200 µl serum-free medium and seeded in the top chambers with or without matrigel coated, and 600 µl complete medium containing 20% FBS was added to the bottom chambers as a chemoattractant. After 24 h, cells penetrating into the bottom chamber were captured using a IX53 fluorescence microscope (Olympus, Tokyo, Japan). In matrigel invasion assays, cells in the bottom chambers were fixed, stained with crystal violet and photographed.

### Apoptosis assay

At 48 h post-transfection, AML cells were collected and subjected to apoptosis analysis using the Annexin V/propidium iodide (PI) dual staining assay (MultiSciences, Hangzhou, CN) on a Navios flow cytometer (Beckman coulter, Miami, FL, USA). Data were analyzed using FlowJo v10 (BD, Ashland, OR, USA).

### Western blotting assay

At 72 h post-transfection, AML cells were harvested and lysed immediately using RIPA Lysis Buffer (Sigma-Aldrich, St.Louis, MO, USA) supplemented with protease inhibitor phenylmethanesulfonylfluoride fluoride (PMSF; Beyotime, Shanghai, CN). Then the protein was determined using antibodies against MSI2 (Clone: EP1305Y, Abcam, Cambridge, UK), DLL1 (Cat#20230-1-AP, Proteintech, Wuhan, CN), HES1 (Clone: D6P2U, Cell Signaling Technology (CST), Beverly, MA, USA), cleaved NOTCH1 (Clone: (D1E11) XP^®^, CST), NANOG (Cat#A3232, ABclonal, Wuhan, CN), OCT4 (Cat#A7920, ABclonal), SOX2 (Cat#A0561, ABclonal), SNAIL1 (Clone: C15D3, CST), MMP2 (Clone: D4M2N, CST), MMP9 (Clone: (D6O3H) XP^®^, CST), β-actin (Cat#AF7018, Effinity) and GAPDH (Cat#60004-1-1 g, Proteintech), at 1/1,000 dilution for incubation overnight at 4 °C, respectively. The chemiluminescence was detected using Fluor Chem E (ProteinSimple, USA) and the quantification of the blots was analyzed with Image J software (NIH, Bethesda, MD, USA).

### RNA immunoprecipitation (RIP)

1 × 10^7^ Lenti-MSI2 vector-transfecting HEL (MSI2-OE. HEL) cells were collected and treated with lysis buffer (RNA immunoprecipitation kit, Geneseed Biotech, Guangzhou, CN) containing 1% protease inhibitor and 1% RNase inhibitor. 10% lysis supernatant served as Input, and 45% lysis supernatant was incubated in immunoprecipitation reactions with 5 μg MSI2 antibody bound to magnetic beads (named Anti-MSI2), and 45% lysis supernatant was incubated with 5 μg IgG antibody (Clone: DA1E, CST) bound to magnetic beads as isotype control (named IgG). RNA was extracted and detected by qRT-PCR as described previously [29].

### RNA-stability measurements

AML cells were treated with actinomycin D (Meilunbio, Dalian, CN) at a final concentration of 5 mg/mL for 0.5, 1, 1.5, 2 or 3 h and harvested for total RNA extraction. Then, the mRNA expression levels of DLL1 and Snail1 were detected using qRT-PCR to analyze their mRNA half-life as described previously [30].

### Animal models

Male 4–6 weeks old NOG mice (NOD.Cg-*Prkdc*^*scid*^*Il2rg*^*tm1Sug*^/JicCrl) and male 4–6 weeks old BALB/c-Nu mice were purchased from Charles River (Beijing, CN) and bred under SPF conditions in the Laboratory Animal Center of the First Affiliated Hospital of Wenzhou Medical University. NOG mice were injected intravenously with 5 × 10^6^ MSI2-OE. HEL cells or NC. HEL cells to establish orthotopic AML xenograft model (*N* = 10 mice per group, 6 mice were used for analysis of survival, and another 4 mice were sacrificed 4 weeks after tumor inoculation for analysis of AML progression). As the subcutaneous tumor xenograft model is easy to dynamically observe the growth status of tumor, the subcutaneous AML xenograft model was built. For BALB/c-Nu mice, cyclophosphamide (CPA; Macklin, Shanghai, CN) at 80 mg/kg was administered intraperitoneally (i.p.) before tumor inoculation. Then a total of 5 × 10^6^ MSI2-OE. HEL cells per mouse were subcutaneously (s.c.) injected into BALB/c-Nu mice with Matrigel matrix (#356234, Corning, Corning, NY, USA) at equal volume, while another group of mice were injected s.c. with 5 × 10^6^ NC. HEL cells. When the tumor size reached about 150–200 mm^3^, mice were grouped randomly (*N* = 7 mice per group) and treated with 3 nmol micrOFFTM agomir-143 or agomir-NC (Sequences shown in Additional file 1: Table S2) intratumorally every three days for two weeks. Tumor volume was recorded every three days and calculated: length × (width)^2^ × 0.5. All of mouse experiments have been approved by the Laboratory Animal Ethics Committee of the First Affiliated Hospital of Wenzhou Medical University and have been performed in accordance with relevant institutions and national guidelines and regulations.

### Statistical analysis

Data were analyzed as mean ± SD from at least three independent replicates with the level of significance defined as *P* < 0.05. The *t*-test was carried out to compare between two groups, and a one-way ANOVA followed by post-hoc test was used to determine the difference between multiple groups. Statistical analyses were performed using GraphPad Prism 8.0 (GraphPad software, San Diego, CA, USA).

## Results

### MiR-143 is lowly expressed in AML and inhibits its development

**Fig. 1:**
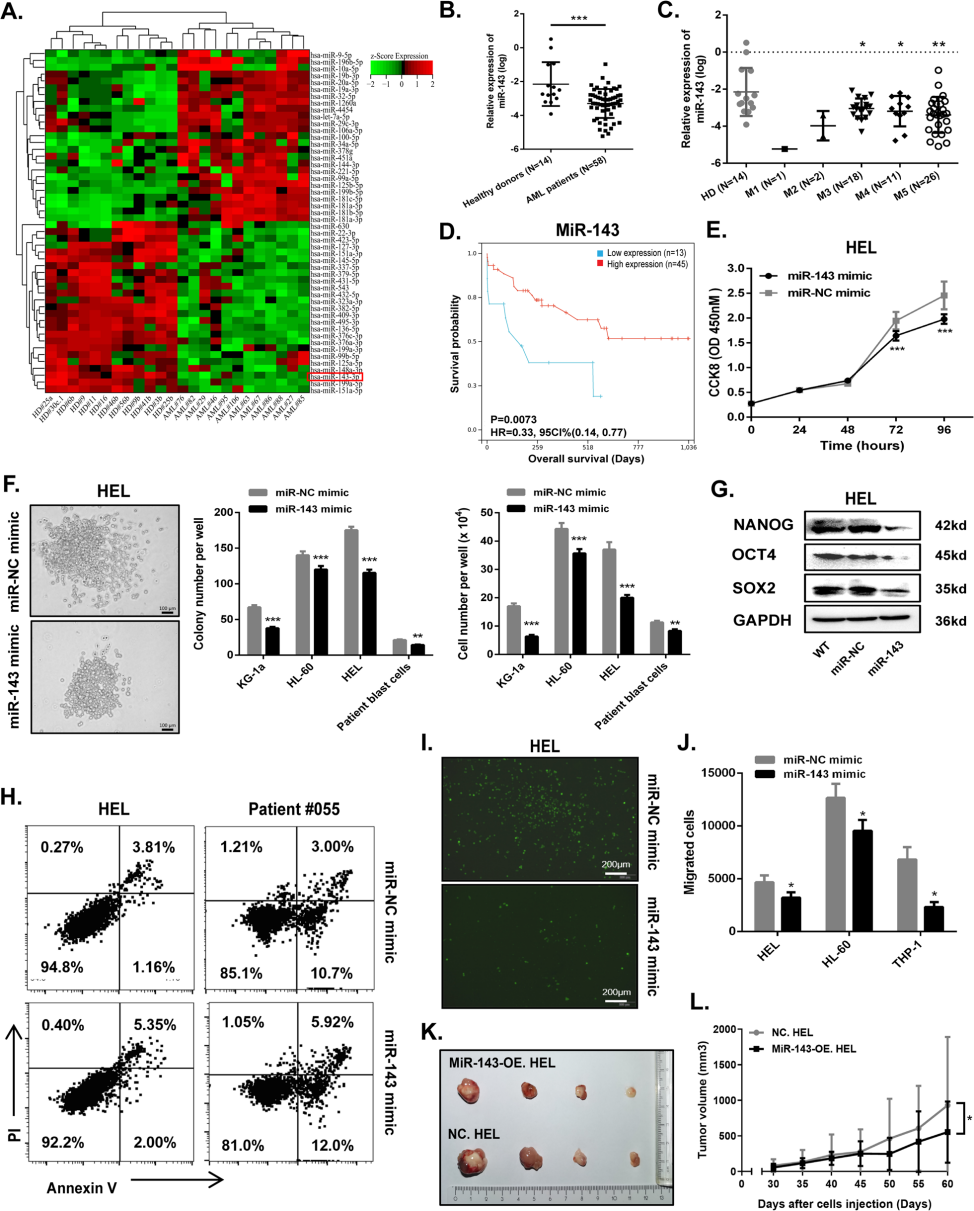
MiR-143 is lowly expressed in AML and inhibits the development of AML. **A** Heatmap of miRNAs (Top 25 of upregulated miRNAs and Top 25 of downregulated miRNAs) in peripheral blood of newly diagnosed cytogenetically normal AML patients (AML; *N* = 12) and normal healthy donors (HD; *N* = 12) from a GEO Dataset (GSE142699). Hsa-miR-143-3p was marked with the red box. (**B**) Relative expression of miR-143 was determined in BM aspirates from healthy donors (N = 14) and newly diagnosed AML patients (N = 58). **C** Relative expression of miR-143 was further exhibited in different FAB subtypes. **D** AML patients were divided into the miR-143 high expression group (≥ 1.863e-4, N = 45) and low expression group (< 1.863e-4, N = 13) according to the optimal cutoff value (1.863e-4) for risk score and the OS of the patients was analyzed (*P* = 0.0073). (**E**) Comparison of cell proliferation in HEL cells transfected with miR-143 or miR-NC mimic by CCK-8 assay. **F** Colony formation in HEL cells decreased after the transfection of miR-143 mimic. Representative images of colonies (left panel) and statistical analysis diagrams of colony number and cell number in various AML cell lines and patient blast cells were shown (right panel), scale bar = 100 μm. (**G**) Cancer stemness-related proteins in HEL cells transfected with miR-143 or miR-NC mimic were determined by Western blotting. **H** Apoptosis of HEL cells and blasts from Patient#055 transfected with miR-143 or miR-NC mimic was assessed by Annexin V and PI staining. **I** Cell migration in EGFP-labeled HEL cells transfected with miR-143 or miR-NC mimic was detected by transwell assay and observed by fluorescence microscope, scale bar = 200 μm. **J** Migration of different AML cells was illustrated by counting cells from lower chambers. (**K**, **L**) BALB/c-Nu mice were subcutaneously transplanted with 3 × 10^6^ MiR143-OE. HEL or NC. HEL cells to establish xenograft AML model. The tumor growth was measured via tumor volume, *N* = 4. Data are expressed as mean ± SD (error bars). * *P* < 0.05, ** *P* < 0.01 and ***P < 0.001, *t*-test

It has been reported that miR-143, as a tumor suppressor, has low expression in a variety of malignant tumors and is closely related to the occurrence, development and prognosis of malignant tumors [31–33]. A heatmap of miRNAs was generated from a GEO dataset (GSE142699) to show that the expression level of miR‐143 was downregulated markedly in newly diagnosed AML patients compared to healthy donors (Fig. 1A), as well as the results shown in our cohort (Fig. 1B, C). And patients with low miR-143 level had shorter overall survival (OS) than those with high miR-143 level (Fig. 1D).

We then transfected AML cells with miR-143 to explore its biological effects (Additional file 1: Fig. S1A). CCK-8 assays showed that miR-143 mimic suppressed the proliferation of AML cell lines HEL and HL-60 (Fig. 1E and Additional file 1: Fig. S1B). Moreover, clonogenic capacity was markedly impaired in miR-143-transfected AML cell lines including HEL, HL-60, and KG-1α, as well as CD34^+^ primary AML cells from patients (Fig. 1F and Additional file 1: Fig. S1C). Further molecular mechanism found that cancer stemness-related proteins including NANOG, OCT4, SOX2 were dramatically downregulated (Fig. 1G and Additional file 1: Fig. S1D). Flow cytometry analysis showed that more apoptosis (Fig. 1H and Additional file 1: Fig. S1E) occurred in all miR-143-transfected AML cells tested, whereas the cell cycle distribution was almost unaffected (Additional file 1: Fig. S2A, B). Additionally, transwell assay revealed that treatment with miR-143 mimic obviously attenuated the migration of AML cell lines including HEL, HL-60 and THP-1 (Fig. 1I, J and Additional file 1: Fig. S2C).

To further confirm the role of miR-143 in AML, we also constitutively overexpressed miR-143 in HEL (MiR143-OE. HEL) cells (Additional file 1: Fig. S3A, B). MiR143-OE.HEL cells had slower proliferation, impaired clonogenic capability, and obvious downregulation of cancer stemness-related proteins compared to NC.HEL cells (Additional file 1: Fig. S3C–F). Meanwhile, MiR143-OE. HEL cells were more prone to apoptosis than NC. HEL cells (Additional file 1: Fig. S3G). In the subcutaneous xenograft mouse model, tumors grew more slowly as they grew larger in the MiR143-OE. HEL group (Fig. 1K, L). Taken together, our data unraveled that miR-143 was lowly expressed and exerted various biological roles including inhibiting the proliferation and clonogenic capability and promoting apoptosis in AML.

### MiR-143 directly binds to MSI2

**Fig. 2:**
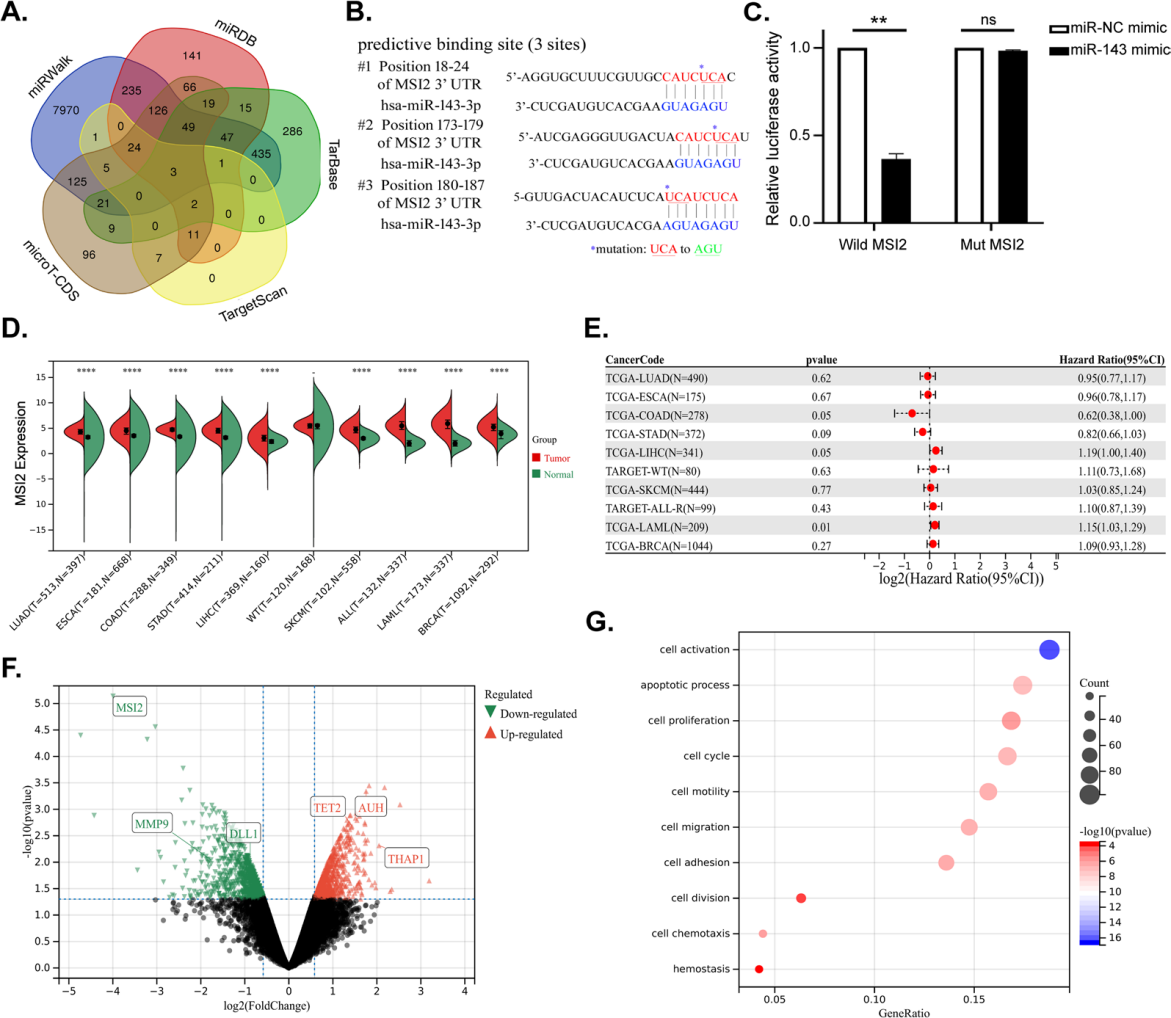
MiR-143 directly binds to MSI2 (**A**) Five bioinformatics prediction softwares (miRWalk, miRDB, TarBase, TargetScan, and microT-CDS) were used to select three target genes (*MSI2*, *VASH1* and *GATM*) that could be interacted with hsa-miR-143-3p. (**B**) Schematic illustration showed complementation in the regions of the 3′UTR of *MSI2* mRNA (#1: positions 18-24, #2:173-179 and #3:180-187) to the mature miR-143. Colored (red and blue) sequences of three sites indicated the predicted binding sites for miR-143. The nucleotide sequence of the mutated site was shown in green. (**C**) Luciferase activities were determined in KG-1α cells co-transfected with miR-143 or miR-NC mimic and wild-type or mutant-type pMIR-MSI2 vectors to verify the predictive miR-143 binding sites in the 3′UTR of MSI2. (**D**) MSI2 expression between normal and tumor samples in log2(x + 0.001) transform from the UCSC database (TCGA, TARGET, and GTEx) was analyzed via Unpaired Wilcoxon Rank Sum and Signed Rank Tests. (**E**) Relationship between MSI2 expression and prognosis in each tumor was analyzed by Cox proportional hazards regression model established by coxph function of the R package survival via Log-rank test. (**F**) Volcano map of DEGs in GSE22775 (upregulated genes were marked in red and downregulated genes were marked in green). (**G**) GO pathway analysis (biological processes) was performed on 621 DEGs

To gain insight into the mechanism of action of miR-143 in AML, we used five online bioinformatics databases and found that *MSI2* mRNA was predicted to have a binding site with miR-143-3p (Fig. 2A). Further analysis using publicly available algorithms predicted that miR-143 might be a potential regulator of MSI2 (Fig. 2B). The 3′UTR of *MSI2* mRNA containing the possible miR‐143 binding sites was cloned into a reporter plasmid. The luciferase activity of wild-type pMIR-MSI2 was suppressed after cotransfection with miR-143 in KG1α cells, and the suppression of luciferase activity could be abrogated when the binding sites were mutated (Fig. 2C).

Moreover, we extracted *MSI2* gene expression data in standardized pan-cancer dataset and analyzed the difference between normal and tumor samples (Fig. 2D), as well as the relationship between gene expression and prognosis in each tumor (Fig. 2E). We found that *MSI2* gene was overexpressed in AML, leading to a poor prognosis. Furthermore, differentially expressed genes (DEGs) from GEO Datasets (GSE22775) were identified, of which 250 genes were upregulated and 371 genes were downregulated when AML cell lines were treated with MSI2 shRNA (Fig. 2F). And GO pathway analyses identified enriched pathways in DEGs, including cell activation, migration, apoptosis and proliferation (Fig. 2G). Therefore, these data suggest that MSI2 is a direct target gene of miR-143 in AML.

### MSI2 is overexpressed in AML and facilitates leukemogenesis

**Fig. 3:**
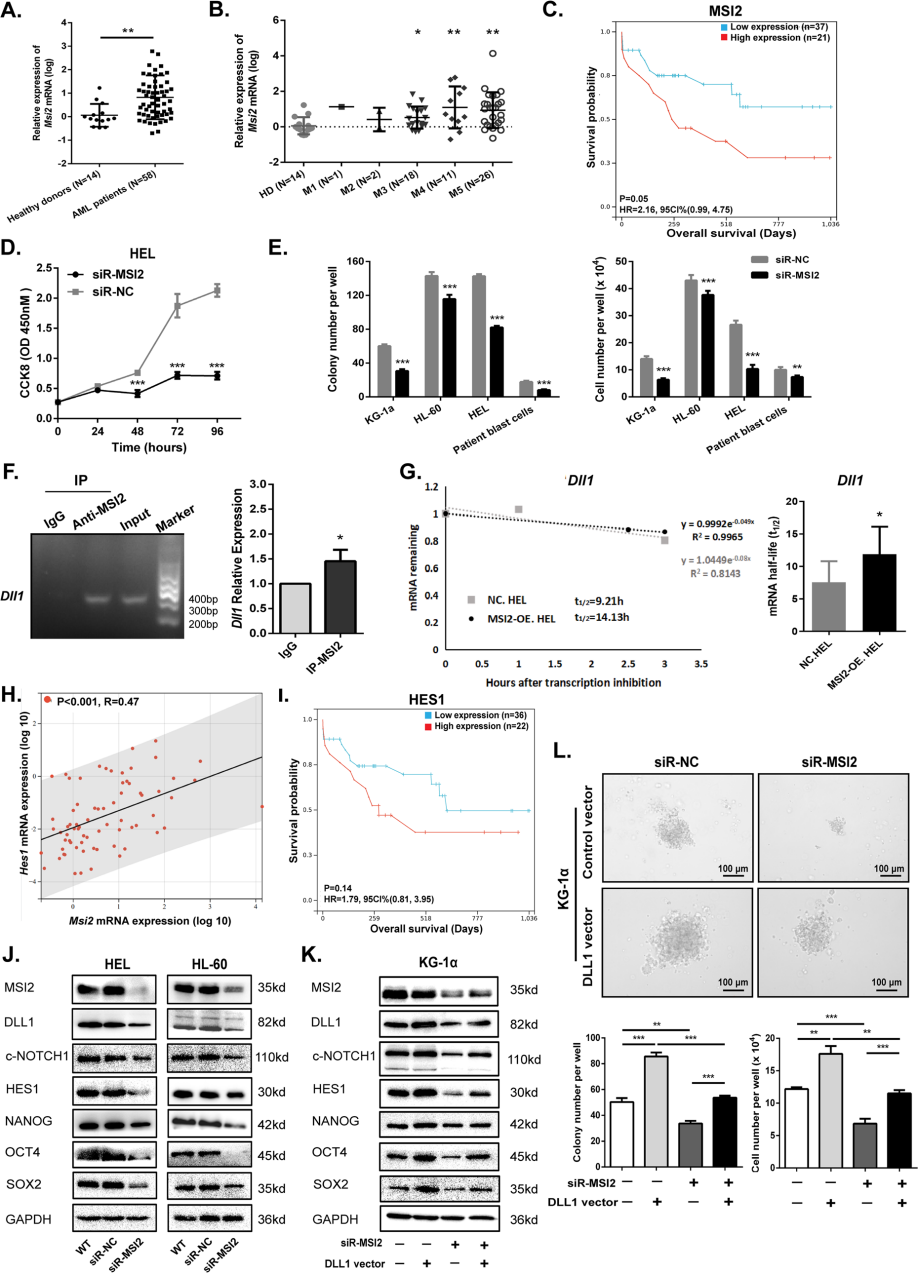
MSI2 is overexpressed in AML and facilitates leukemogenesis (**A**) Relative expression of MSI2 mRNA was determined in BM aspirates from healthy donors (N = 14) and newly diagnosed AML patients (N = 58). (**B**) Relative expression of MSI2 mRNA was exhibited in different FAB subtypes. (**C**) AML patients were divided into the MSI2 high expression group (≥ 11.861, N = 21) and low expression group (< 11.861, N = 37) according to the optimal cutoff value (11.861) for risk score and the OS of the patients was observed (P = 0.05). (**D**) Effect of MSI2 knockdown using siRNAs on the cell growth was detected in HEL cells by CCK-8 assay. (**E**) The clonogenic capacity of KG-1α, HL-60, HEL cells and patient AML blasts was weakened after siR-MSI2 transfection. (**F**) Agarose gel electrophoresis (left panel) and RIP-PCR (right panel) were used to test the interaction between MSI2 protein and *DLL1* mRNA. *P < 0.05 vs. IgG. (**G**) Half-life (t_1/2_) of *DLL1* transcripts in MSI2-OE. HEL and NC. HEL cells were shown in exponential regression curves (left panel) and statistical analysis diagram (right panel). (**H**) Relative expression of *MSI2* and *HES1* mRNA was determined in BM aspirates from healthy donors (N = 14) and newly diagnosed AML patients (N = 58). Correlation between *MSI2* mRNA expression and *HES1* mRNA expression was assessed (*P* < 0.001, R = 0.47, *N* = 72). **(I)** AML patients were divided into the HES1 high expression group (≥ 0.062, *N* = 22) and low expression group (< 0.062, *N* = 36) according to the optimal cutoff value (0.062) for risk score and the OS of the patients was observed (*P* = 0.14). (**J**) Core components of Notch1 signaling including NOTCH1 ligand (DLL1), cleaved NOTCH1 (c-NOTCH1) and HES1, and cancer stemness-related proteins including NANOG, OCT4 and SOX2 were determined in HEL and HL-60 cells transfected with siR-MSI2 or siR-NC by western blot. (**K**) Key factors of Notch1 signaling pathway and cancer stemness-related proteins were determined in Lenti- DLL1 vector- or Lenti- Control vector-transfecting KG-1α cells after the transfection with siR-MSI2 or siR-NC. (**L**) Colony formation was performed in Lenti- DLL1 vector- or Lenti- Control vector-transfecting KG-1α cells after the transfection with siR-MSI2 or siR-NC. Representative plots (above) and statistical analysis diagram (below) were illustrated. Data are expressed as mean ± SD (error bars). * *P* < 0.05, ** *P* < 0.01 and ***P < 0.001, *t*-test

Then we verified that *MSI2* mRNA level in newly diagnosed AML patients was higher than that in healthy donors (Fig. 3A, B). Moreover, patients with higher level of *MSI2* had a trend of shorter OS than those with lower level of *MSI2* (Fig. 3C), indicating that MSI2 is an oncogene in human AML. To identify the functional characterization of MSI2, siRNAs were designed to knock down MSI2 (Additional file 1: Fig. S4A). AML cells transiently transfected with siR-MSI2 proliferated more slowly as the concentration of siRNA increased (Additional file 1: Fig. S4B, C) and the treatment time prolonged (Fig. 3D). Moreover, silencing of MSI2 significantly impaired the clonogenic capacity (Fig. 3E and Additional file 1: Fig. S4D) and accelerated apoptosis (Additional file 1: Fig. S4E) in three AML cell lines including KG-1α, HL-60, HEL, as well as primary AML cells. On the contrary, forced expression of MSI2 led to a significant increase of cell proliferation, colony formation and a decrease of cell apoptosis in HEL cells (Additional file 1: Fig. S5A–F). In brief, MSI2 contributed to the maintenance of stemness in AML cells and thereby promoted leukemogenesis.

### Positive feedback regulation of MSI2/DLL1/Notch1 signaling for AML development and maintenance

HITS-CLIP previously showed that DLL1 might be a direct downstream target of MSI2, which can regulate the self-renewal and differentiation of hematopoietic stem cells by activating Notch signaling [15]. Our RIP-PCR test showed a 1.46-fold higher level of *DLL1* mRNA in lysis supernatant from MSI2-OE. HEL cells incubated with anti-MSI2 antibody compared to that with anti-IgG antibody, indicating that MSI2 as a RBP has a close interaction with *DLL1* mRNA (Fig. 3F). And the overexpression of MSI2 caused an increase in the half-life of *DLL1* transcripts (Fig. 3G). Furthermore, we identified that the expression level of MSI2 was positively correlated with that of HES1, a downstream target of Notch1 signaling with a trend towards worse prognosis (Fig. 3H, I).

So, we next explored whether MSI2 functions through the Notch1 signaling pathway. We observed that the key factors of Notch1 signaling pathway and cancer stemness-related proteins were downregulated when MSI2 is silenced (Fig. 3J) and upregulated when it is overexpressed (Additional file 1: Fig. S5G). In KG-1α cells, overexpression of DLL1 could partially reverse the biological effect caused by knockdown of MSI2 through restoring the expression levels of the key factors of Notch1 signaling pathway and cancer stemness-related proteins (Fig. 3K and Additional file 1: Fig. S5H). In addition, the clonogenic capacity was significantly increased in DLL1 vector transfecting KG-1α cells, but it was weakened after MSI2 silencing (Fig. 3L). Conclusively, these data suggest that MSI2 regulates the clonogenic capacity of AML cells by DLL1/Notch1 signaling.

### MSI2 regulates AML cell migration through post-transcriptional control of Snail1

**Fig. 4:**
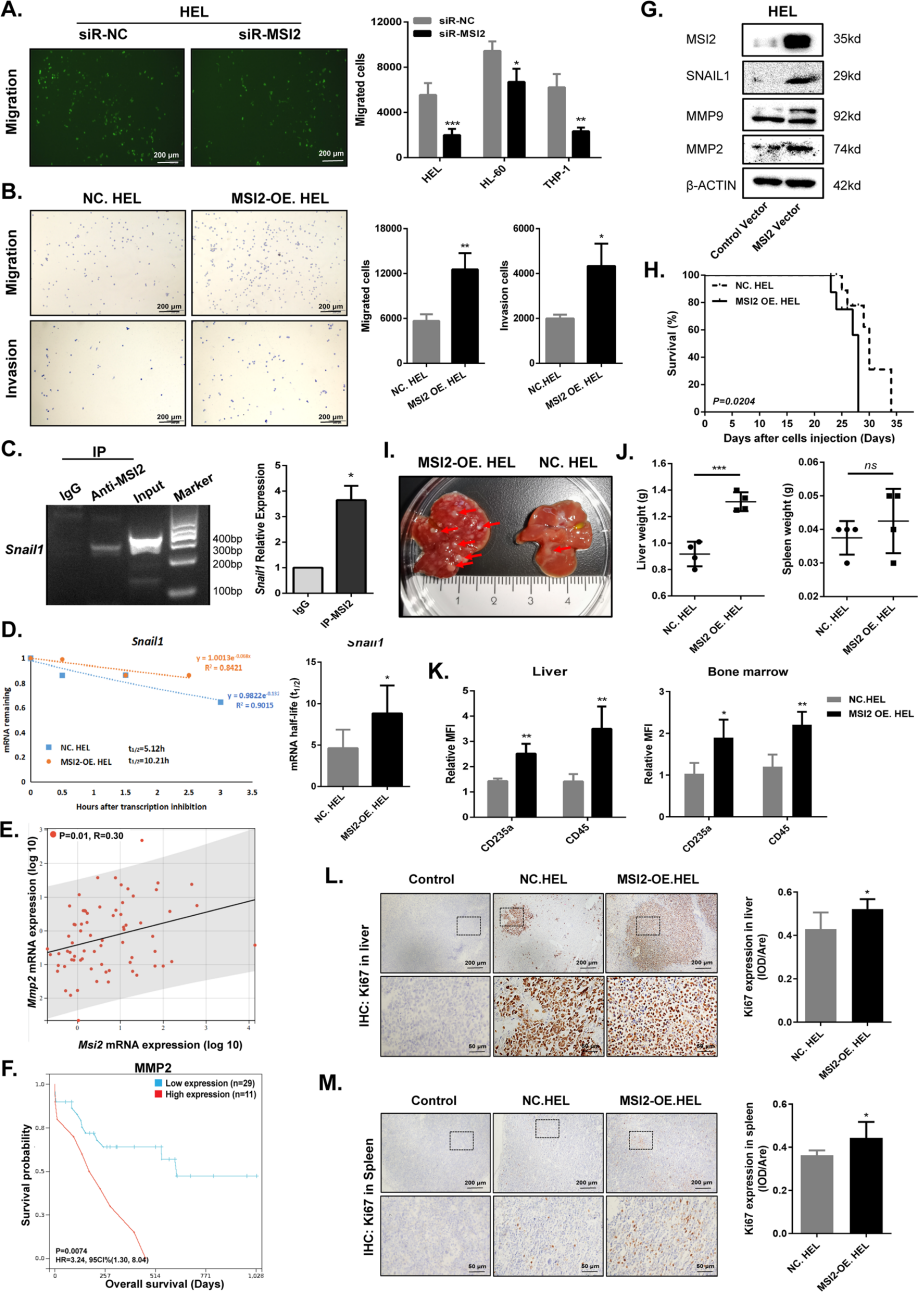
MSI2 exerts the metastatic properties in AML. **A** Cell migration in EGFP-labeled HEL cells transfected with siR-MSI2 or siR-NC was detected by transwell assay. Representative pictures (left panel) from three independent experiments were observed by fluorescence microscope and statistical analysis diagram (right panel) was illustrated by counting cells from lower chambers, scale bar = 200 μm. **B** Cell migration and invasion increased in MSI2-OE. HEL cells compared to those in NC. HEL cells. Cells from lower chambers of transwell were counted and stained with crystal violet, scale bar = 200 μm. Representative pictures (left panel) and statistical analysis diagram (right panel) were illustrated. **C** Agarose gel electrophoresis (left panel) and RIP-PCR (right panel) were used to test the interaction between the MSI2 protein and *Snail1* mRNA. *P < 0.05 vs. IgG. **D** *Snail1* mRNA stability curves (left panel) were plotted as qRT-PCR expression with time in NC. HEL and MSI2-OE. HEL cells. Half-life (t_1/2_) was calculated from the stability curves in statistical analysis diagram (right panel). **E** Relative levels of *MSI2* and *MMP2* mRNA were determined in BM aspirates from healthy donors (N = 14) and newly diagnosed AML patients (N = 58). Correlation between *MSI2* mRNA expression and *MMP2* mRNA expression was assessed (*P* = 0.01, R = 0.30, *N* = 72). **F** AML patients (excluding AML-M3 subtype) were divided into the MMP2 high expression group (≥ 1.563, *N* = 11) and low expression group (< 1.563, *N* = 29) according to the optimal cutoff value (1.563) for risk score and the OS of the patients was observed (*P* = 0.0074). (**G**) Expression of MSI2 and tumor metastasis-associated proteins in Lenti- MSI2 vector- or Lenti- Control vector-transfecting HEL cells. **H** NOG mice were intravenously transplanted with 5.0 × 10^6^ NC.HEL or MSI2-OE. HEL cells. Kaplan–Meier survival curve was shown, *P* = 0.0204, *N* = 6, log-rank test. **I** Nodules (red arrow) appeared on the livers from two groups of mice. (**J**) Liver weight (left panel) and spleen weight (right panel) from two groups of mice were measured.* N* = 4, *t*-test. **K** Relative mean fluorescence intensity (MFI) of CD235a and CD45 in liver (left panel) and bone marrow (right panel) from two groups of mice. **L**, **M** Expression of Ki67 in liver (**L**) and spleen (**M**) was detected by IHC. Representative pictures (left panel) and statistical analysis diagram (right panel) were illustrated. Data are expressed as mean ± SD (error bars). * *P* < 0.05, ** *P* < 0.01 and ***P < 0.001, *t*-test

As GO pathway analyses showing the identified enriched pathways including cell migration in DEGs that was associated with downregulation of MSI2 (Fig. 2G), transwell assays were performed to determine the roles of MSI2 on migration. We observed that less migration occurred in AML cells transfected with siR-MSI2 (Fig. 4A and Additional file 1: Fig. S6A), while overexpression of MSI2 promoted the migratory and invasive abilities of HEL cells (Fig. 4B).

Then, RIP followed by qRT-PCR was conducted to explore migration-related proteins downstream of MSI2. The expression level of Snail1 was 3.65-fold higher than the control, indicating that the MSI2 could physically bind to *Snail1* mRNA (Fig. 4C and Additional file 1: Fig. S6B). And we made time-course RNA decay curves for *Snail1* mRNA and found that the half-life of *Snail1* transcripts was longer in MSI2-OE. HEL cells than that in NC. HEL cells (Fig. 4D), showing that MSI2 functioned to stabilize the *Snail1* mRNA. Accordingly, we selected common downstream proteins regulated by Snail1 and identified that the expression level of MSI2 was positively correlated with that of MMP2 in AML patients (Fig. 4E), with the findings that MMP2 was overexpressed in AML (Additional file 1: Fig. S6C, D) and had a poor prognosis in AML patients excluding acute promyelocytic leukemia (Fig. 4F). Consistently, we observed obvious upregulation of Snail1, MMP2 and MMP9 protein followed by the overexpression of MSI2 (Fig. 4G and Additional file 1: Fig. S6E).

Additionally, we established an in vivo xenograft model to further validate the effect of MSI2 in accelerating metastasis. Compared with the control group, NOG mice intravenously injected with MSI2-OE. HEL cells developed paralysis earlier (Additional file 1: Fig. S7A) and had a shorter survival (Fig. 4H) with more white blood cells and less red blood cells (Additional file 1: Fig. S7B, C). As shown in Fig. 4I, more nodules appeared on the livers of MSI2-OE. HEL group mice, leading to a heavier liver weight (Fig. 4J). Based on the above phenomenon, we took the liver, spleen and bone marrow from mice and found that more human antigens were expressed in the MSI2-OE. HEL group (Fig. 4K–M and Additional file 1: Fig. S7D–G, S8), indicating that overexpression of MSI2 can promote hematopoietic tumor cells to infiltrate into other organs and tissues.

### MSI2 activity is negatively modulated by miR-143 in vitro

**Fig. 5:**
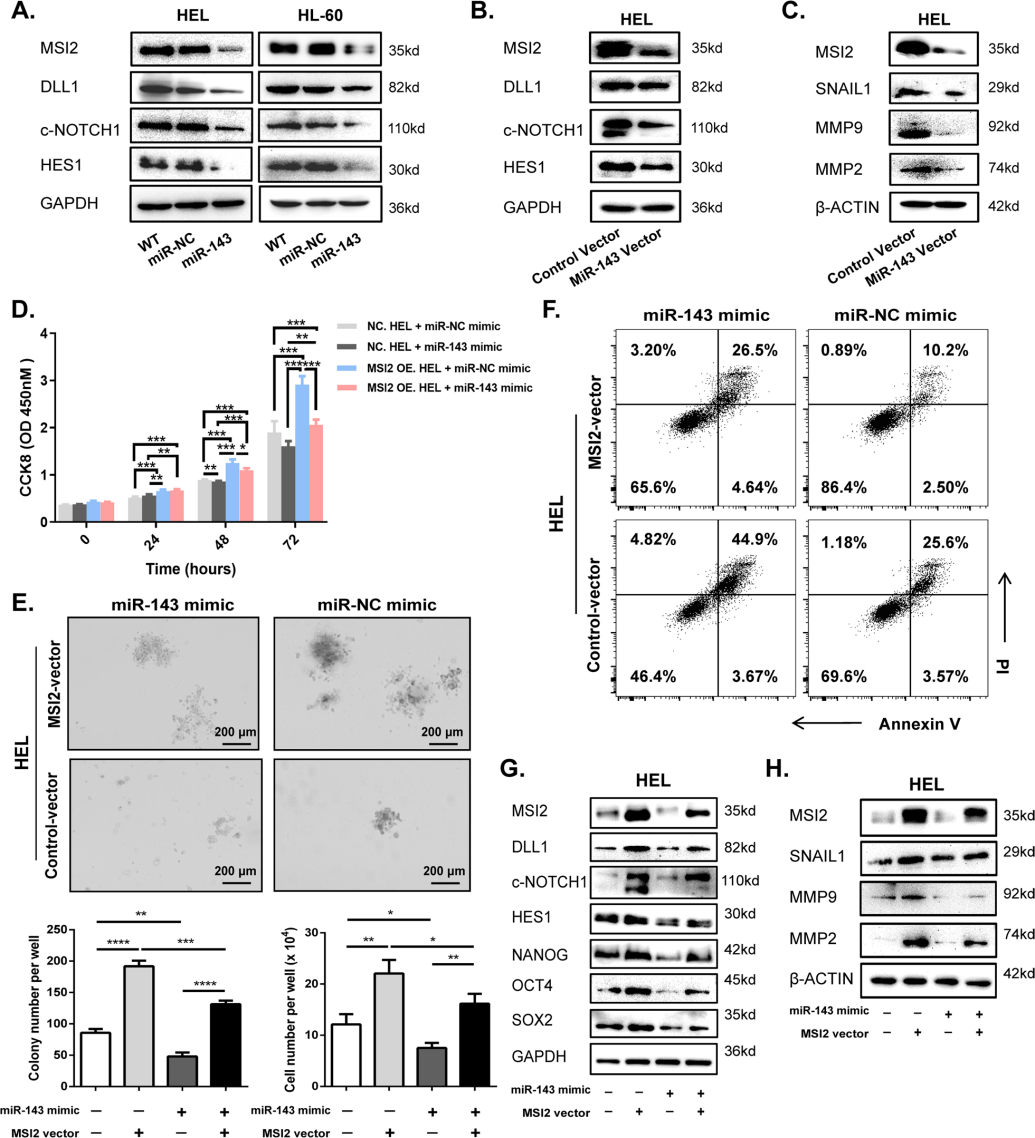
MSI2 activity is negatively modulated by miR-143 in vitro (**A**) Key factors of Notch1 signaling pathway were determined in HEL and HL-60 cells transfected with miR-143 or miR-NC mimic. (**B**, **C**) Key factors of Notch1 signaling pathway (B) and tumor metastasis-associated proteins (C) were measured in Lenti- MiR-143 vector- or Lenti-Control vector-transfecting HEL cells. (**D**) Cell growth of MSI2-OE. HEL and NC. HEL cells transfected with miR-143 or miR-NC mimic was analyzed by CCK-8 assay. (**E**) Clonogenic capacity of Lenti-MSI2 vector- or Lenti-Control vector-transfecting HEL cells after the transfection with miR-143 or miR-NC mimic. Representative pictures (above) and statistical analysis diagram (below) were illustrated, scale bar = 200 μm. (**F**) Apoptosis in Lenti-MSI2 vector- or Lenti-Control vector-transfecting HEL cells transfected with miR-143 or miR-NC mimic. (**G**, **H**) Expression of MSI2, key factors of Notch1 signaling pathway and cancer stemness-related proteins (**G**) and tumor metastasis-associated proteins (**H**) were shown in Lenti-MSI2 vector- or Lenti-Control vector-transfecting HEL cells transfected with miR-143 or miR-NC mimic. Data are expressed as mean ± SD (error bars). * *P* < 0.05, ** *P* < 0.01 and ***P < 0.001, *t*-test

To determine whether miR-143 works as a tumor suppressor to reverse the carcinogenic effects of MSI2, western blotting assays were performed to verify that the expression of key factors of Notch1 signaling pathway were affected negatively by miR-143 mimic in HEL and HL-60 cells, as well as migration-relative proteins in MiR-143 vector-transfecting HEL cells (Fig. 5A–C).

Moreover, we found that MSI2-OE. HEL cells grew faster than NC. HEL cells, but their proliferation slowed down when miR-143 was upregulated (Fig. 5D). And miR-143 inhibited MSI2’s role of promoting colony formation and maintaining stemness in MSI2-vector-transfecting HEL and KG-1α cells (Fig. 5E, Additional file 1: Fig. S9A, B), as well as reversing the anti-apoptotic effect of MSI2 (Fig. 5F). Identically, reduced protein levels were shown in MSI2-overexpressing AML cells when they were transfected with miR-143, indicating that miR-143 to some extent prevented MSI2 from activating the Notch1 signaling pathway and cancer stemness-related genes (Fig. 5G and Additional file 1: Fig. S9C–E), and it also partially reversed the expression of migration-related proteins regulated by MSI2 (Fig. 5H and Additional file 1: Fig. S9F).

### MiR-143 inhibits the carcinogenic effect of MSI2 in vivo

**Fig. 6:**
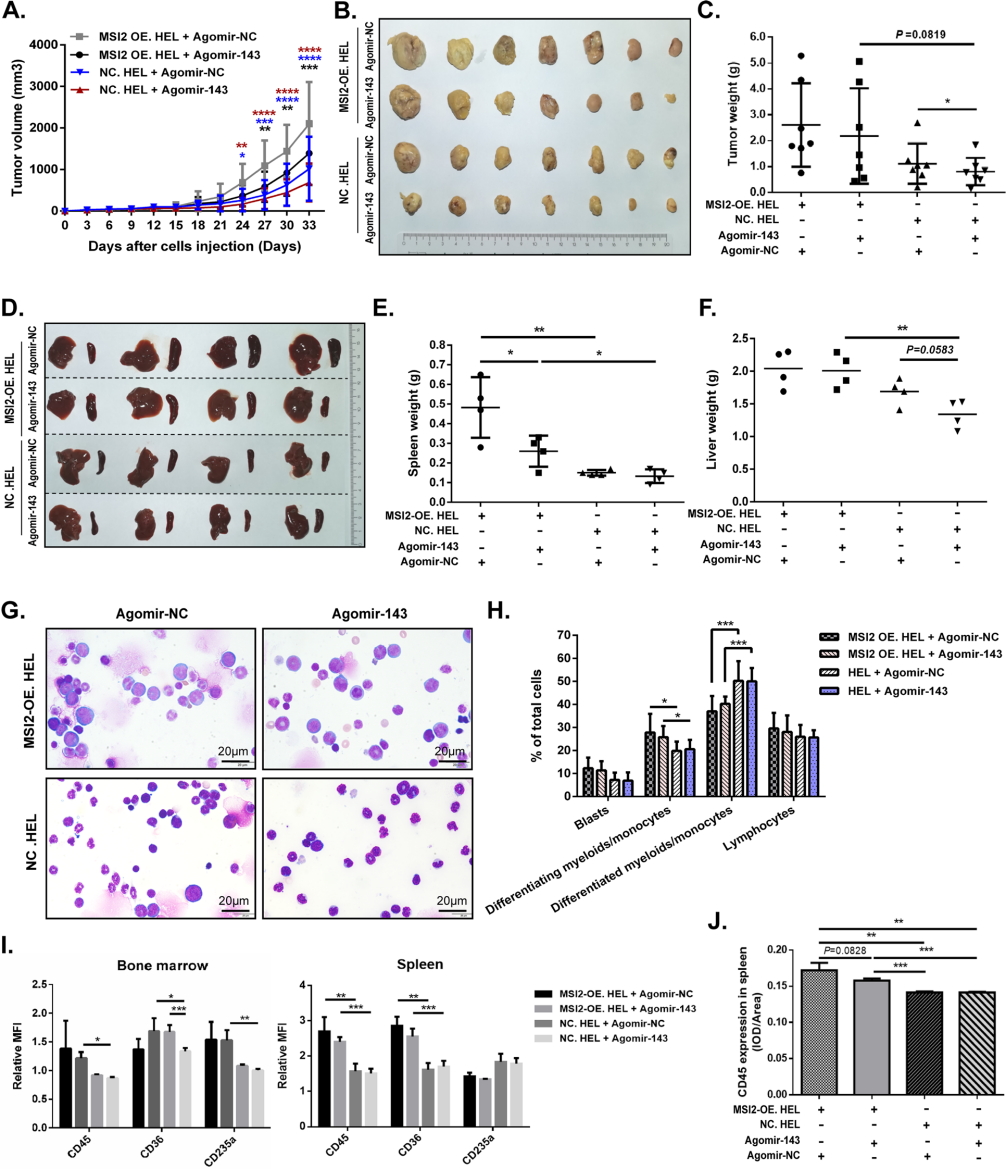
MiR-143 inhibits the carcinogenic effect of MSI2 in vivo (**A**) BALB/c-Nu mice were subcutaneously inoculated with 5 × 10^6^ MSI2-OE. HEL cells or NC. HEL cells to establish xenograft AML model, and then treated intratumorally with micrOFFTM agomir-143 or agomir-NC. Tumor volumes from each group of mice were recorded regularly, *N* = 7. (**B**) Tumors from four groups of mice were presented respectively, *N* = 7. (**C**) Tumor weights were recorded, *N* = 7. (**D–F**) Spleens and livers from four groups of mice were presented (**D**), and spleen weight (**E**) and liver weight (F) were measured, *N* = 4. (**G**) Representative Wright-Giemsa staining of bone marrow cells from each group of mice at the end point, scale bar = 20 μm. (**H**) Proportion of bone marrow cells, including blasts, differentiating myeloids/monocytes, differentiated myeloids/monocytes and lymphocytes. (**I**) Relative MFI of CD45, CD36 and CD235a in bone marrow (left panel) and spleen (right panel) from each group of mice. (**J**) Expression of CD45 in spleen from each group of mice was detected by IHC. Data are expressed as mean ± SD (error bars). * *P* < 0.05, ** *P* < 0.01 and ***P < 0.001, *t*-test

To investigate whether miR-143 exerts an inhibitory effect on MSI2’s promoting leukemia progression in vivo, subcutaneous xenograft AML mouse model was established and treated intratumorally with agomir-143 for preventing tumor growth. We found that tumor grew faster in the MSI2-OE. HEL group than those in the NC. HEL group (Fig. 6A). And 33 days after tumor inoculation, each group of mice was euthanized and their tumor, liver, spleen and BM were dissected. We found that larger sizes and heavier weights of tumor (Fig. 6B, C) and spleen and liver (Fig. 6D–F) appeared in the MSI2-OE. HEL group. Those results revealed the oncogenic role of MSI2 in vivo. On the other hand, the tumor growth in either MSI2-OE. HEL group or NC. HEL group was partially inhibited in the treatment with agomir-143, as well as size and weight of spleen and liver (Fig. 6A–F).

To assess and compare the degree of AML infiltration between these groups, BM and spleen tissues from mice were ground for analysis. As expected, Wright—Giemsa of BM smears revealed that there were more blasts and differentiating myeloids/monocytes in the MSI2-OE. HEL group (Fig. 6G, H). Consistently, flow cytometric analysis and IHC showed that the expression of human antigens in BM, and spleen was higher in the MSI2-OE. HEL group (Fig. 6I, J, Additional file 1: Fig. S10, 11), indicating that the subcutaneous tumor inoculated with MSI2-OE. HEL cells invaded and infiltrated into BM and spleen tissues, which further proved that MSI2 promoted the leukemia progression in vivo. Moreover, human CD45 in BM or spleen expressed slightly lower in the agomir-143-treated group than those in the agomir-NC-treated group, illustrating that miR-143 partially inhibited tumor growth and metastasis in vivo. Taken together, these data suggest that miR-143 inhibits the carcinogenic effect of MSI2 in AML.

## Discussion

RBPs, a group of proteins that participate in the formation of ribonucleoprotein complexes by binding pre-mRNAs and mRNAs, regulate the fate of a RNA post-transcriptionally by the way of RNA splicing, modification, nuclear export, localization, stability and translation [34]. Recent studies have found that abnormal expression of RBPs is ubiquitously associated with the promotion of cancer progression through co- and post-transcriptional mechanisms [3].

Musashi-1 and MSI2 as RBPs have been found to be overexpressed in various cancer [10, 35–38], as well as our current results that MSI2 was highly expressed in AML patients, leading to poor prognosis. And we demonstrated that MSI2 possessed properties of self-renewal, migration and invasion, in turn leading to the progression of AML. Nevertheless, the molecular mechanisms involved in MSI2 maintaining these properties in AML remains to be elucidated.

Consistent with MSI2’s known functions in regulating the Notch signaling pathway, DLL1 was found among the high-confidence MSI2 targets by HITS-CLIP [39]. We found that MSI2 bound directly to *DLL1* mRNA at the post-transcriptional level to activate its downstream Notch1 signaling pathway by regulating the stability of *DLL1* mRNA. As an agonistic ligand for Notch receptors, DLL1 is overexpressed in AML with poor prognosis (Additional file 1: Fig. S12A, B) and triggers proteolytic cleavage of Notch receptors, and the resulting Notch intracellular domain can be translocated into the nucleus and interacted with CSL to activate the transcription of target genes, such as *CCND1*, *HES1* and *MYC*, which in turn promotes tumor cell proliferation and survival [40, 41]. Growing evidence has showed that Notch signaling pathway functions to regulate the self-renewal and differentiation of hematopoietic stem cells [42]. In accordance with these previous studies, we found that knockdown of MSI2 could inhibit the expression of DLL1, thereby attenuating the stemness-related gene expression and clonogenic capacity in AML cells. However, overexpression of DLL1 activated Notch1 signaling pathway and rescued the stemness properties.

As GO pathway analyses identified cell migration pathway in AML, our study indicated a functional relevance of MSI2 in regulating AML cell migration through post-transcriptional control of Snail1 by regulating the stability of *Snail1* mRNA. Snail1, as a transcriptional repressor, is overexpressed in AML and causes poor prognosis in AML from standardized pan-cancer dataset (Additional file 1: Fig. S12C, D). It has been reported that Snail1 upregulates MMP2 and MMP9 to trigger EMT, and the synergistic effect of Snail1 and Slug maintains EMT through continuous stimulation of MMP9 [43, 44]. In our experiment, MSI2 knockdown significantly inhibited the migration of AML cells, while overexpression of MSI2 showed the opposite effect by upregulating Snail1 protein level and subsequent expression of MMPs, which confirmed our hypothesis that MSI2 facilitated migration of AML cells by activating the MSI2/Snail1/MMPs axis. Besides, our animal experiments found that mice injected with MSI2-OE. HEL cells developed hindlimb paralysis earlier and more nodules in the liver, indicating that MSI2 overexpression boosts more leukemia cells to infiltrate into other organs and tissues.

Recently, approaches to inhibiting MSI2 proteins based on RNA interference have achieved promising outcomes in preclinical research [4, 8]. Zheng et al. employed weighted correlation network analysis to produce a lncRNA-miRNA-mRNA ceRNA network in gastric cancer from 12 both cancer-associated and prognosis-related genes, where MSI2 was paired with hsa-miR-143-3p [45]. We used five bioinformatics prediction softwares (miRWalk, miRDB, TarBase, TargetScan, and microT-CDS) to find that *MSI2*, *vasohibin-1* (*VASH1)* and *glycine amidinotransferase (GATM)* mRNA were predicted to have a binding site with miR-143-3p. VASH1 mediated miR-143-induced cell dissemination and angiogenesis [25], while GATM encodes a mitochondrial enzyme and its mutation often causes mitochondrial-related diseases [46]. Our previous study has revealed that MSI2 silencing exerts potent activity against AML [47]. And as expected, binding of miR-143 to the 3′-UTR of *MSI2* mRNA was validated by luciferase reporter assay, providing evidence that miR-143 reversed the role of MSI2 in AML development. Pramanik et al. demonstrated that restitution of tumor suppressor microRNAs using a systemic nanovector could inhibit pancreatic cancer growth in mice [48]. Consistently, our in vivo experiments showed that subcutaneous AML tumors grew more slowly and less leukemia cells infiltrated into spleen and BM after intratumoral treatment with nanoparticle-encapsulated miR-143, demonstrating that miR-143 functioned as a tumor suppressor to prevent AML progression. However, the partial rescue may be due to the effect that miR-143 is likely to bind to multiple genes on leukemogenesis.

## Conclusions

**Fig. 7:**
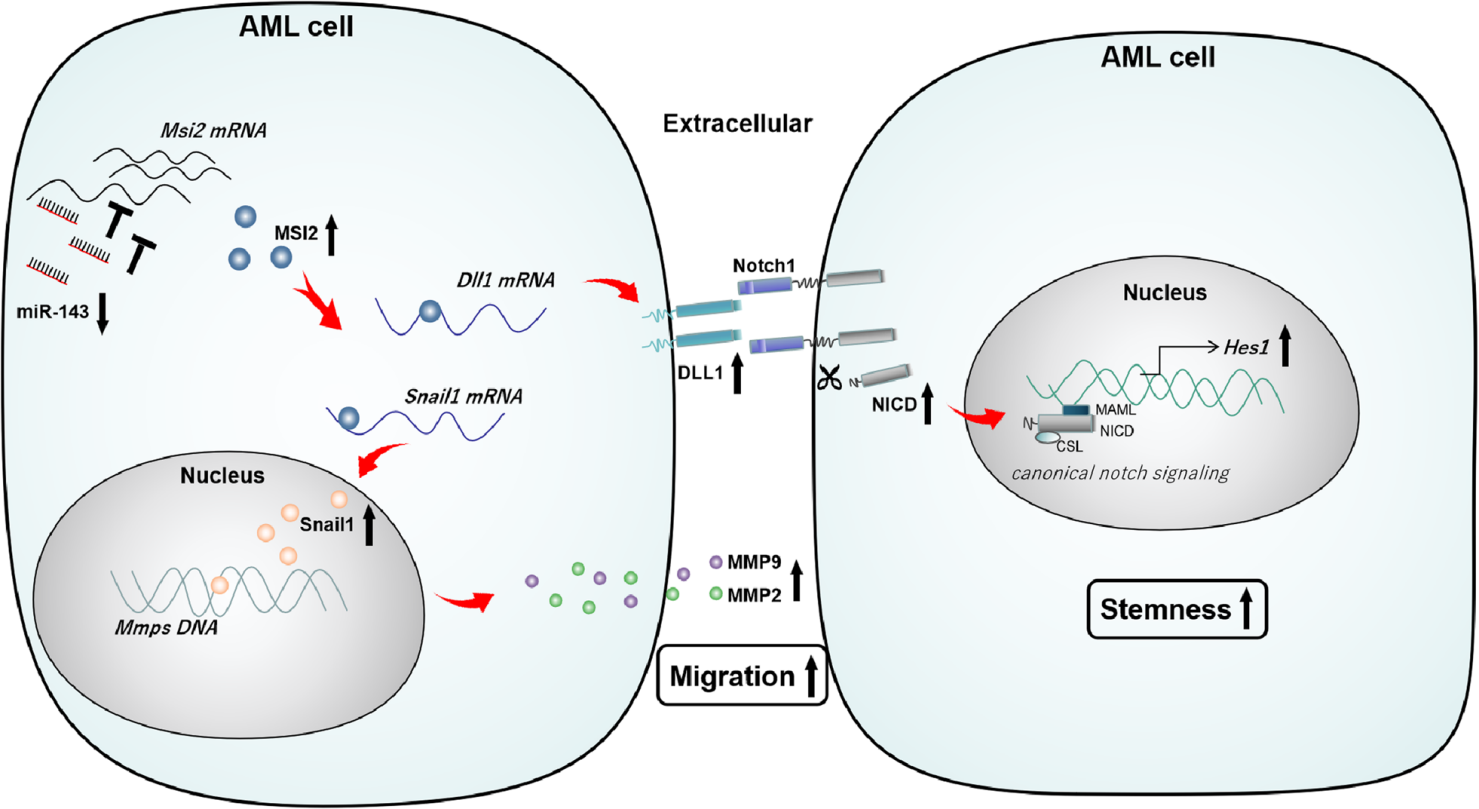
Schematic illustrates the mechanism by which miR-143 directly binds to MSI2 and inhibits its efficacy in AML progression. MSI2/DLL1/Notch1 axis: MSI2 binds directly to *DLL1* mRNA and stabilize it at the post-transcriptional level. Then DLL1 agonistic ligands trigger proteolytic cleavage of Notch1 receptors to generate the NICD. NICD/CSL-dependent transcriptional activation of target gene* HES1* is upregulated by canonical Notch signaling cascades to maintain stemness of AML cells. MSI2/Snail1/MMPs axis: MSI2 stabilizes Snail1 mRNA and induced transcriptional upregulation of Snail1, which in turn activated both MMP2 and MMP9 to promote AML cell migration. AML, Acute myeloid leukemia; MiR-143, MicroRNA-143; MSI2, Musashi-2; DLL, delta-like canonical Notch ligand; NICD, Notch intracellular domain; MAML, mastermind like protein; CSL, cBF1-suppressor of hairless-LAG1; HES1, hes family bHLH transcription factor 1; Snail1: snail family transcriptional repressor 1; *MMP* matrix metalloproteinase

Our research has revealed a novel mechanism (Fig. 7) by which MSI2 exerts its pro-proliferative properties via the MSI2/DLL1/Notch1 cascade and metastatic properties via the MSI2/Snail1/MMPs axis in AML, and miR-143 as a negative regulator of MSI2 can serve as a tumor suppressor to predict prognosis and develop personalized treatment strategies for AML patients.

## Supplementary Information

**Additional file 1: Table S2.** Sequences of siRNA/miRNA. **Table S3.** Sequences of primers used for quantitative real-time PCR. **Figure S1.** Biological effects of miR-143 in AML. **Figure S2.** Overexpression of MiR-143 hardly unaffected the cell cycle, but suppressed cell migration in AML. **Figure S3.** Overexpression of MiR-143 inhibited cell growth and induced cell apoptosis in AML. **Figure S4.** Biological effects of MSI2 in AML. **Figure S5.** Overexpression of MSI2 promoted growth and inhibited apoptosis in AML. **Figure S6.** Migration effect of MSI2 in AML. **Figure S7.** Flow cytometric analysis for human antigen expression in mouse tissues. **Figure S8.** IHC analysis for human antigen expression in mouse tissues. **Figure S9**. MSI2 activity was negatively modulated by miR-143 in AML cells. **Figure S10.** Expression of CD45 in spleen was detected by IHC. **Figure S11**. Flow cytometric analysis for human antigen expression. **Figure S12**. *DLL1* and *Snail1* gene expression in clinic.

**Additional file 2:** **Table S1.** Baseline characteristics of participants.

## Data Availability

The data supporting the conclusions of this article are provided in this article and the Additional files. In addition, all data from this study can be obtained from the corresponding author upon reasonable request.

## Abbreviations

MSI2: Musashi-2
AML: Acute myeloid leukemia
MiR-143: MicroRNA-143
MMP: Matrix metalloproteinase
HSPC: Hematopoietic stem/progenitor cells
FAB: French-American-British
EMT: Epithelial-mesenchymal transition
ATCC: American type culture collection
FBS: Fetal bovine serum
QRT-PCR: Quantitative real-time polymerase chain reaction
CFU: Colony-forming unit
PI: Propidium iodide
PMSF: Phenylmethanesulfonyl fluoride
CPA: Cyclophosphamide
RBP: RNA-binding protein
RNP: Ribonucleoprotein
NICD: Notch intracellular domain
IHC: Immunohistochemistry
