# Supplementary material for: MicroRNA-143 acts as a tumor suppressor through Musashi-2/DLL1/Notch1 and Musashi-2/Snail1/MMPs axes in acute myeloid leukemia
Source: J Transl Med. 2023 May 6;21:309. doi: 10.1186/s12967-023-04106-6 (PMC10164318; doi:10.1186/s12967-023-04106-6)
Supplement: Supplementary file 1 — Additional file 1: Table S2. Sequences of siRNA/miRNA. Table S3. Sequences of primers used for quantitative real-time PCR. Figure S1. Biological effects of miR-143 in AML. Figure S2. Overexpression of MiR-143 hardly unaffected the cell cycle, but suppressed cell migration in AML. Figure S3. Overexpression of MiR-143 inhibited cell growth and induced cell apoptosis in AML. Figure S4. Biological effects of MSI2 in AML. Figure S5. Overexpression of MSI2 promoted growth and inhibited apoptosis in AML. Figure S6. Migration effect of MSI2 in AML. Figure S7. Flow cytometric analysis for human antigen expression in mouse tissues. Figure S8. IHC analysis for human antigen expression in mouse tissues. Figure S9. MSI2 activity was negatively modulated by miR-143 in AML cells. Figure S10. Expression of CD45 in spleen was detected by IHC. Figure S11. Flow cytometric analysis for human antigen expression. Figure S12. DLL1 and Snail1 gene expression in clinic. [file 12967_2023_4106_MOESM1_ESM.docx]

**Supplementary Methods**

**Lentivirus-mediated overexpression and RNA interference (RNAi)**

*MSI2* gene (GenBank: NM_138962) and *Pre-MicroRNA 143* gene (miRBase: MI0000459) sequences were respectively cloned into *pCDH* vector with GFP expression cassette (*pCDH-GFP*) to obtain the lentiviral expression plasmids. Then *pCDH-MSI2-GFP* and *pCDH-pre-MicroRNA 143-GFP* plasmids were transfected into HEK-293T cells with packaging plasmids for lentiviral particles using polyethylenimine (Sigma, MO, USA), respectively. The culture supernatant was harvested after 72 h and infected HEL cells with 10 μg/mL polybrene (Sigma). After 24 h post-infection, the medium was replaced with fresh growth medium containing 5 μg/mL puromycin (Gibco) for 48 h to screen for Lenti-MSI2 vector-transfecting HEL cells (MSI2-OE. HEL) and Lenti- miR-143 vector-transfecting HEL cells (MiR143-OE. HEL). Lenti-empty vector-transfecting HEL cells (NC. HEL) served as negative control. Transient MSI2 knockdown was performed via transfection of MSI2 siRNA or hsa-miR-143-3p mimic (GenePharma, Shanghai, CN) by Lipofectamine® RNAiMAX Reagent (Invitrogen). Success of lentivirus transfection was investigated via flow cytometry for GFP expression. RNA interference was identified by quantitative polymerase chain reaction (qPCR) analysis. Sequences of siRNA and miRNA were shown in Table S1.

**Database analysis**

We extracted the GEO dataset (GSE142699), in which peripheral blood from newly diagnosed cytogenetically normal acute myeloid leukemia patients (*N*=12) and healthy donors (*N*=12) was obtained for miRNA analysis. And we used five online bioinformatics databases (TargetScan, TarBase, miRDB, miRWalk and microT-CDS) to select target genes that could be combined with hsa-miR-143-3p. Furthermore, we downloaded the unified and standardized pan-cancer dataset from the UCSC (https://xenabrowser.net/) database (TCGA, TARGET, GTEx) (PANCAN, *N*=19131, *G*=60499), and further we extracted ENSG00000153944 (*Msi2*), ENSG00000198719 (*Dll1*) and ENSG00000124216 (*Snail1*) gene expression data in each sample. Log2(x+0.001) transformation was performed on each expression value. R software was used to calculate the differences in expression between normal and tumor samples and establish a Cox proportional hazard regression model to analyze the relationship between gene expression and prognosis in each tumor. Unpaired Wilcoxon Rank Sum and Signed Rank Tests were used for expressive significance and Log-rank test was used for prognostic significance. For gene set functional enrichment analysis, we extracted GEO datasets (GSE22775) and used R software package clusterProfiler (version 3.14.3) to perform gene differential expression analysis between the two groups (|logFC|≥1.0 and *P* < 0.05) and identify deferentially expressed genes (DEGs), including the upregulated genes and downregulated genes. Then DEGs were annotated using Gene Ontology (GO) databases to obtain enriched pathways.

**Flow cytometric assay**

After mice were euthanized, spleen tissues were dissected and milled into single cells and bone marrow was removed from femur. Then cells were incubated with the premixed antibody cocktail as following: Krome Orange-conjugated anti-human CD45, PE-conjugated anti-human CD235a, APC conjugated anti-human CD36 (Beckman Coulter). The isotype-matched IgG1 was used as a negative control.

**Immunohistochemistry**

Spleen and liver tissues from euthanized mice were paraffin-embedded and cut into 4 μm sections. Sections were stained with anti-human CD45 antibody (Clone: D9M8I XP®, CST) or anti-human Ki67 antibody (Clone: SP6, Abcam) and then followed by goat anti-rabbit IgG secondary antibody conjugated with streptavidin-HRP (Biosharp) to detect the metastatic ability of AML xenograft. Protein expression levels were analyzed by calculating the integrated optical density per stained area (IOD/area) using Image J software.

| **Supplementary Tables**  **Table S1. Baseline characteristics of participants (Shown in Additional file 2)**  **Table S2. Sequences of siRNA/miRNA** | | |
| --- | --- | --- |
|  | **Sense (5’-3’)** | **Antisense (5’-3’)** |
| siRNA-MSI2 | GAGAAAGUCUGUGAGAUUCAUTT | AUGAAUCUCACAGACUUUCUCTT |
| siRNA-NC | UUCUCCGAACGUGUCACGUTT | ACGUGACACGUUCGGAGAATT |
| hsa-miR-143-3p mimic | UGAGAUGAAGCACUGUAGCUC | / |
| miR-NC mimic | UUGUACUACACAAAAGUACUG | / |
| micrOFFTM agomir-143 | UGAGAUGAAGCACUGUAGCUC | GCUACAGUGCUUCAUCUCAUU |
| micrOFFTM agomir-NC | UUCUCCGAACGUGUCACGUTT | ACGUGACACGUUCGGAGAATT |

| **Table S3. Sequences of primers used for quantitative real-time PCR** | | | |
| --- | --- | --- | --- |
|  | **Forward primer (5’-3’)** | | **Reverse primer(5’-3’)** |
| MSI2 | | CCACCTCCACACTGCTTCATTCTG | CTCTGTTCCTGCGTGCCACTTG |
| DLL1 | | CAACCTCCCTATCTACACACTG | CATCCTGAGTTTCTCTCTCTCG |
| Snail | | AATACCTCAGCCTGGGTGCC | ATCCTGAGCAGCCGGACTCT |
| HES1 | | AACACTGATTTTGGATGCTCTG | CACTGTCATTTCCAGAATGTCC |
| MMP2 | | ATTGTATTTGATGGCATCGCTC | ATTCATTCCCTGCAAAGAACAC |
| MMP9 | | CAGTACCGAGAGAAAGCCTATT | CAGGATGTCATAGGTCACGTAG |
| GAPDH | | GGGCATCTTGGGCTACACT | GGTCCAGGGTTTCTTACTCC |
| hsa-mir-143-3p | | CTGGCGTTGAGATGAAGCAC | CAGAGCAGGGTCCGAGGTA |
| U6 snRNA | | CGCTTCGGCAGCACATATAC | TTCACGAATTTGCGTGTCATC |

**Supplementary Figures**

**Figure S1**

**
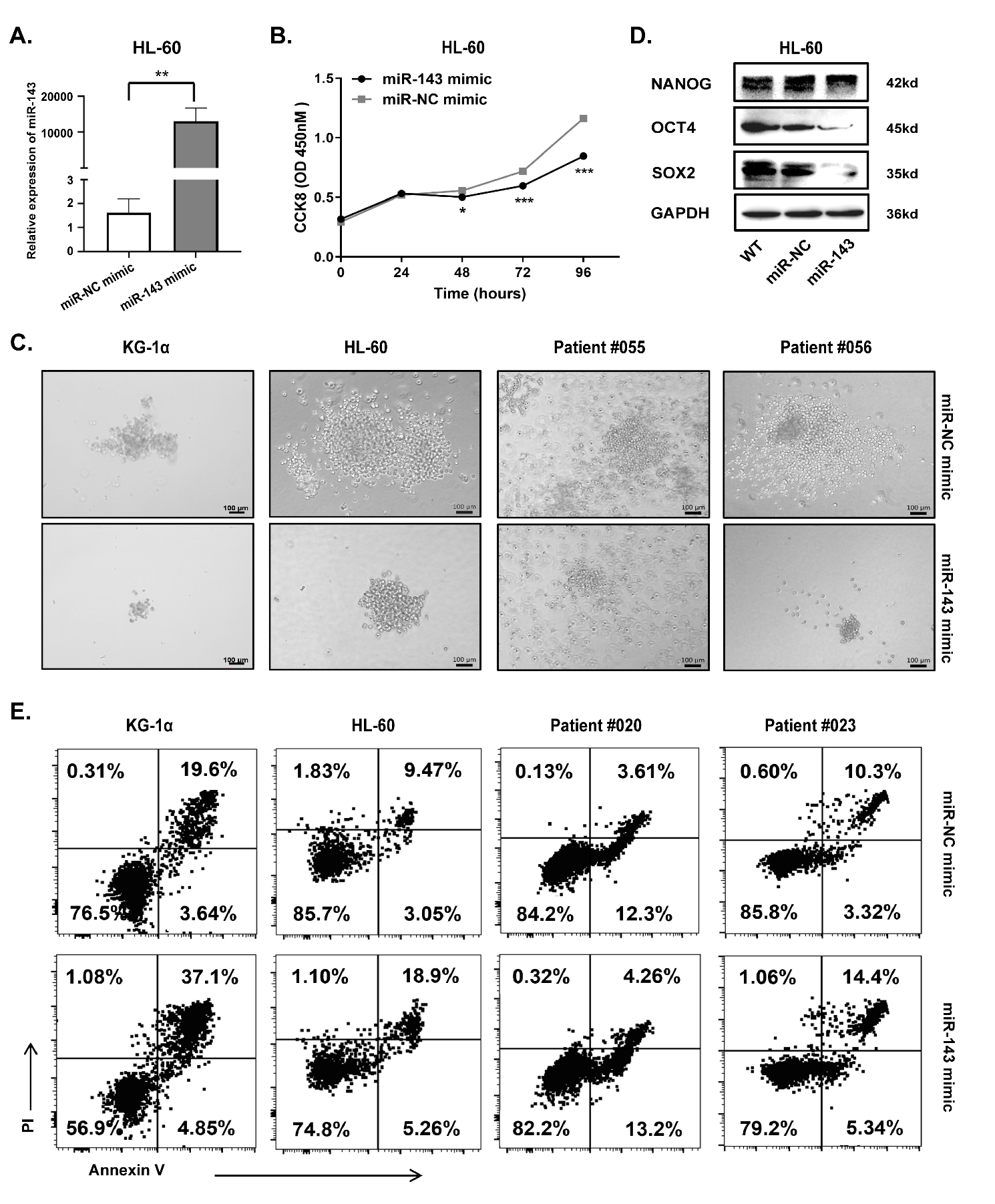
**

**Figure S1. Biological effects of miR-143 in AML**.

**(A)** Effect of miR-143 mimic was verified via qRT-PCR in HL-60 cells. **(B)** Comparison of cell proliferation in HL-60 cells transfected with miR-143 or miR-NC mimic by CCK-8 assay. **(C)** Representative images of colonies in KG-1α, HL-60 and primary AML cells from Patient #055 and #056 transfected with miR-143 or miR-NC mimic, scale bar = 100 μm. **(D)** Cancer stemness-related proteins in HL-60 cells transfected with miR-143 or miR-NC mimic were tested by Western blotting. **(E)** MiR-143 overexpression induced apoptosis in KG-1α, HL-60 and AML blast cells from Patient #020, #023. Data are expressed as mean ± SD (error bars). * *P* < 0.05, ** *P* < 0.01 and ****P* < 0.001, *t*-test.

**Figure S2**


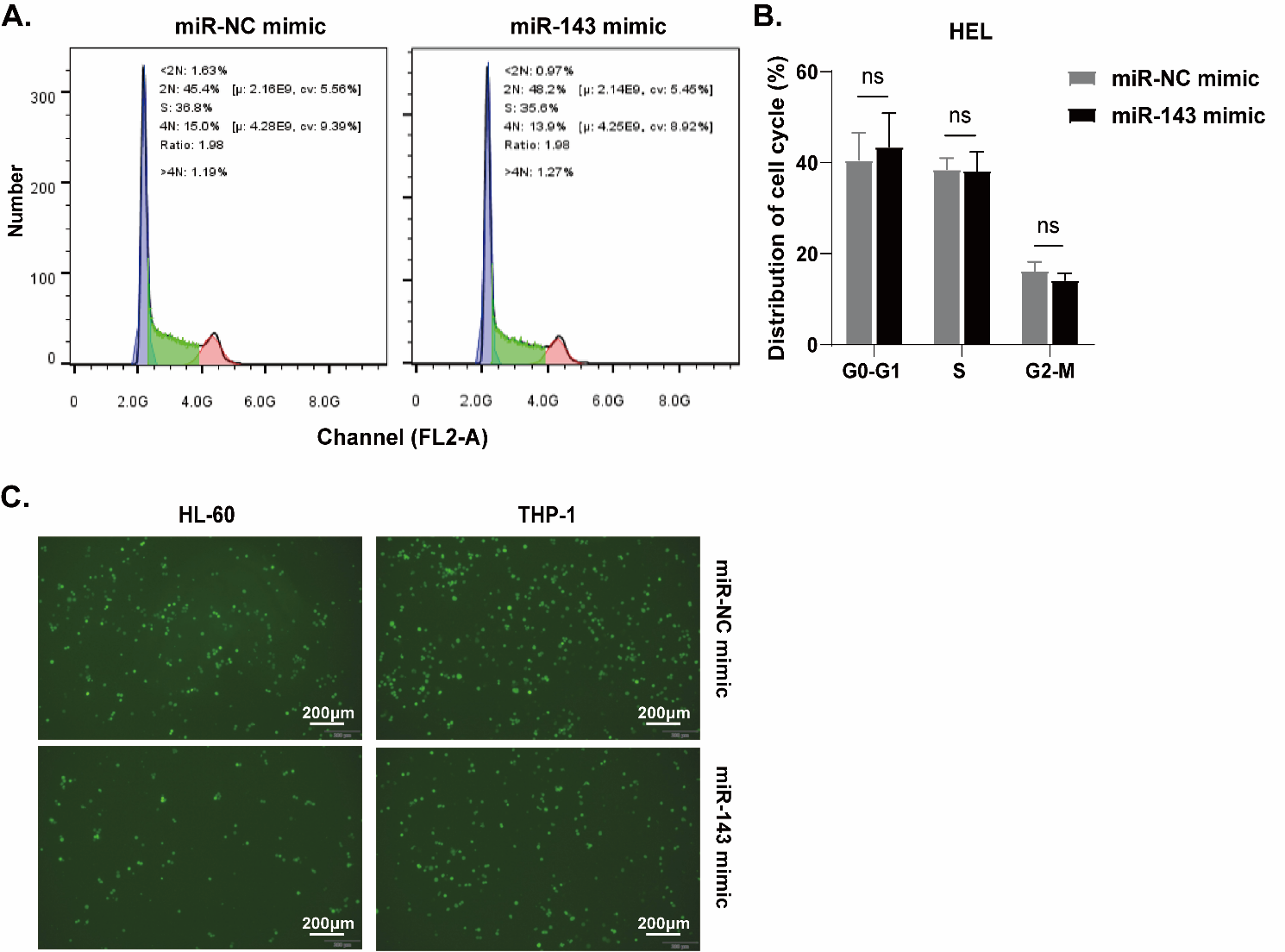


**Figure S2. Overexpression of MiR-143 hardly unaffected the cell cycle, but suppressed cell migration in AML.**

**(A, B)** Cell cycle was tested by flow cytometry in HEL cells transfected with miR-143 or miR-NC mimic. Representative pictures (A) from three independent experiments and statistical analysis diagram (B) were illustrated. Ns: no significance. **(C)** Cell migration in EGFP-labeled HL-60 and THP-1 cells transfected with miR-143 or miR-NC mimic was detected by transwell assay. Representative pictures from three independent experiments were observed by fluorescence microscope, scale bar = 200 μm.

**Figure S3**

**
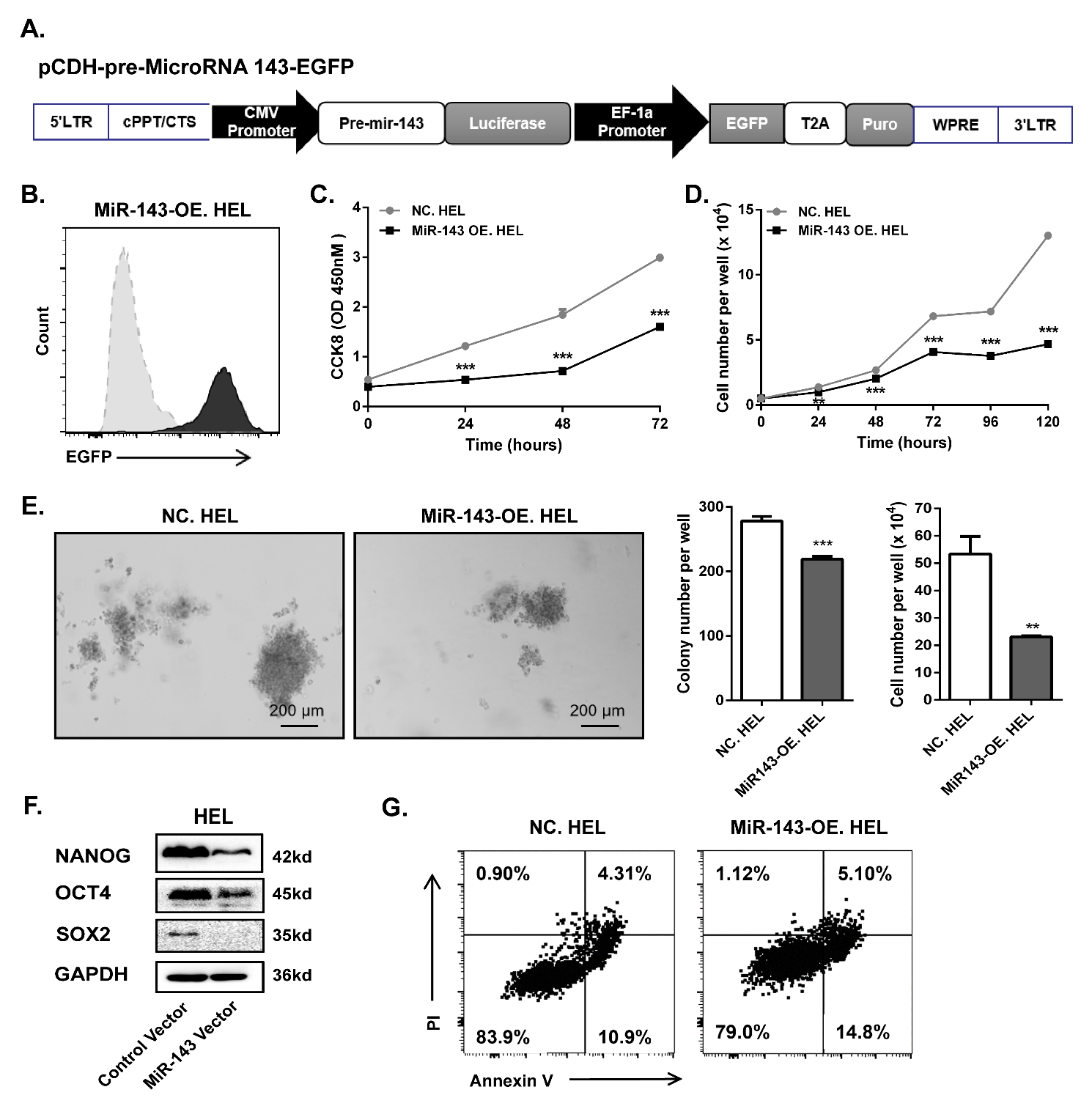
**

**Figure S3. Overexpression of MiR-143 inhibited cell growth and induced cell apoptosis in AML**. **(A)** Schematic illustration of lentiviral expression plasmids. *Pre-MicroRNA-143* gene (miRBase: MI0000459) sequence was inserted into the lentiviral plasmid *pCDH* upstream of an EGFP marker. **(B)** EGFP expression in MiR143-OE. HEL to verify the success of lentivirus transfection. **(C, D)** Growth of MiR143-OE. HEL and NC. HEL cells were evaluated by CCK8 assay (C) and counting (D). **(E)** Colony-forming capacity of NC. HEL cells and MiR143-OE. HEL cells. Representative pictures (left panel) from three independent experiments and statistical analysis diagram (right panel) were illustrated, scale bar = 200 μm. **(F)** Cancer stemness-related proteins in Lenti- MiR-143 vector- or Lenti- Control vector-transfecting HEL cells were tested by Western blotting. **(G)** Apoptosis of MiR143-OE. HEL and NC. HEL cells.

**Figure S4**

**
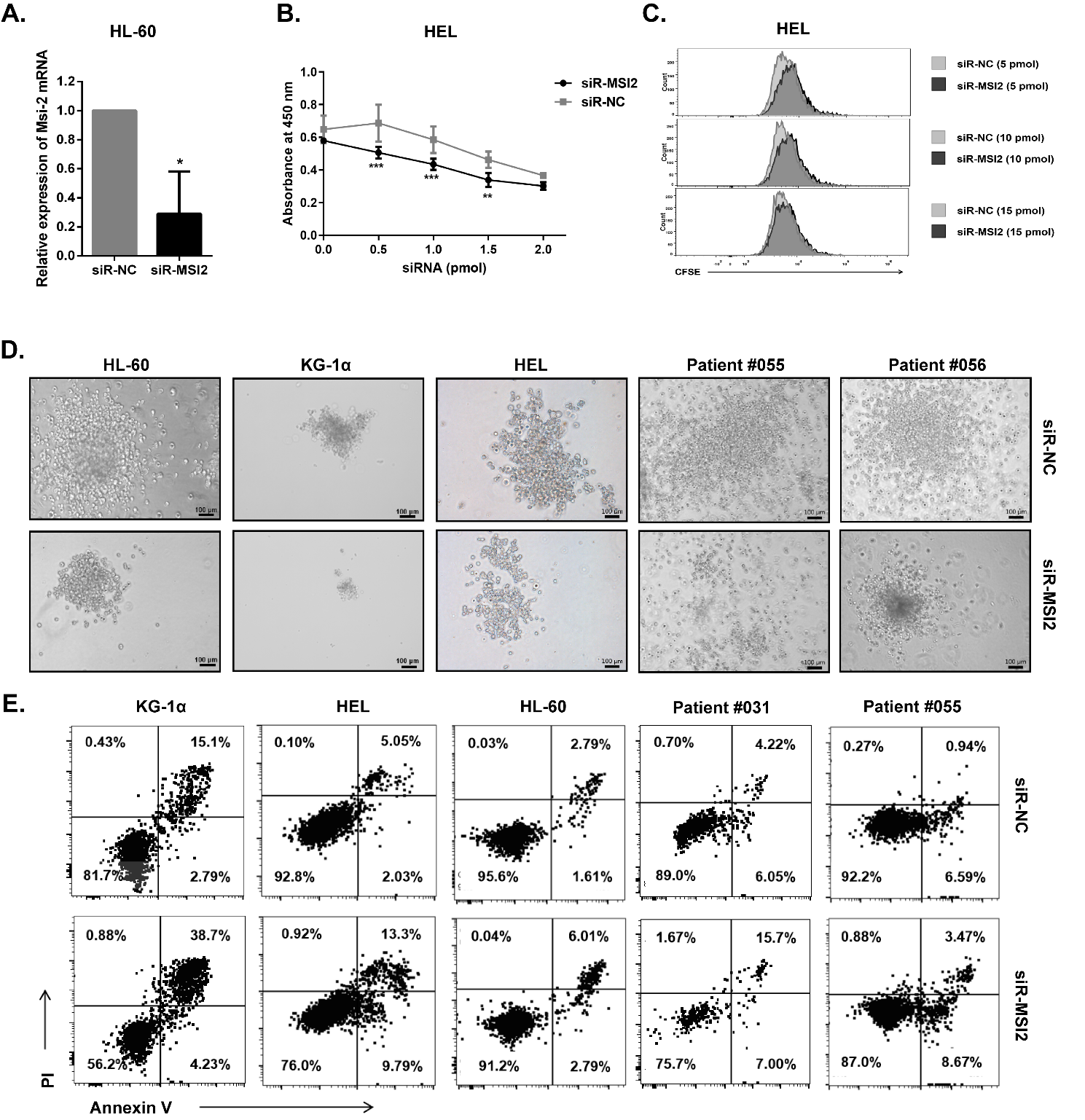
**

**Figure S4. Biological effects of MSI2 in AML**.

**(A)** Effect of siRNA on the interference of *Msi2* mRNA expression in HL-60 was verified by qRT-PCR. **(B, C)** HEL cells were transfected with increasing concentration of siRNA and was detected by CCK-8 assay (B) and CFSE staining (C) at 24 h. **(D)** Representative images of colonies in HL-60, KG-1α, HEL cells and primary AML blast cells from Patient #055 and #056 transfected with siR-MSI2 or siR-NC, scale bar = 100 μm. **(E)** Apoptosis of KG-1α, HEL, HL-60 cells and primary AML blast cells from Patient #031 and #055 transfected with siR-MSI2 or siR-NC was assessed by flow cytometry. Data are expressed as mean ± SD (error bars). * *P* < 0.05, ** *P* < 0.01 and ****P* < 0.001, *t*-test.

**Figure S5**

**
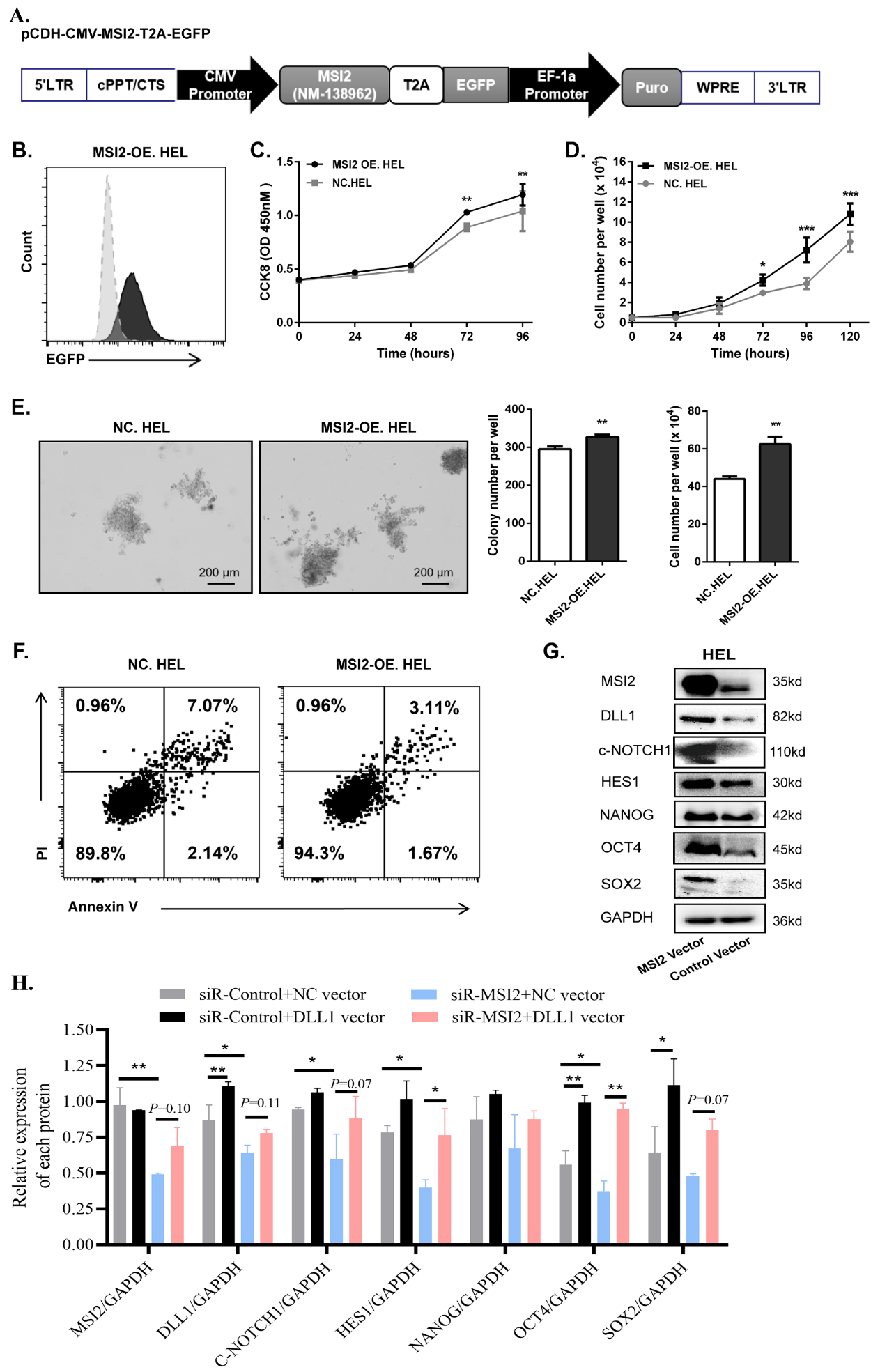
**

**Figure S5. Overexpression of MSI2 promoted growth and inhibited apoptosis in AML**.

**(A)** Schematic illustration of lentiviral expression plasmids. *Msi2* gene (GenBank: NM_138962) sequence was inserted into the lentiviral plasmid *pCDH* upstream of an EGFP marker. **(B)** EGFP expression was verified for the success of lentivirus transfection. **(C, D)** Cell growth of MSI2-OE. HEL and NC. HEL cells were assessed by CCK8 assay (C) and counting (D). **(E)** Colony-forming capacity of MSI2-OE. HEL and NC. HEL cells. Representative pictures (left panel) from three independent experiments and statistical analysis diagram (right panel) were illustrated, scale bar = 200 μm. **(F)** Apoptosis in MSI2-OE. HEL and NC. HEL cells. **(G)** Expression of MSI2, key factors of Notch1 signaling pathway and cancer stemness-related proteins in Lenti-MSI2 vector- or Lenti-Control vector-transfecting HEL cells. **(H)** Relative expression of MSI2, key factors of Notch1 signaling pathway and cancer stemness-related proteins in Lenti- DLL1 vector- or Lenti- Control vector-transfecting KG-1α cells after the transfection with siR-MSI2 or siR-NC. Data are expressed as mean ± SD (error bars). * *P* < 0.05, ** *P* < 0.01 and ****P* < 0.001, *t*-test.

**Figure S6**


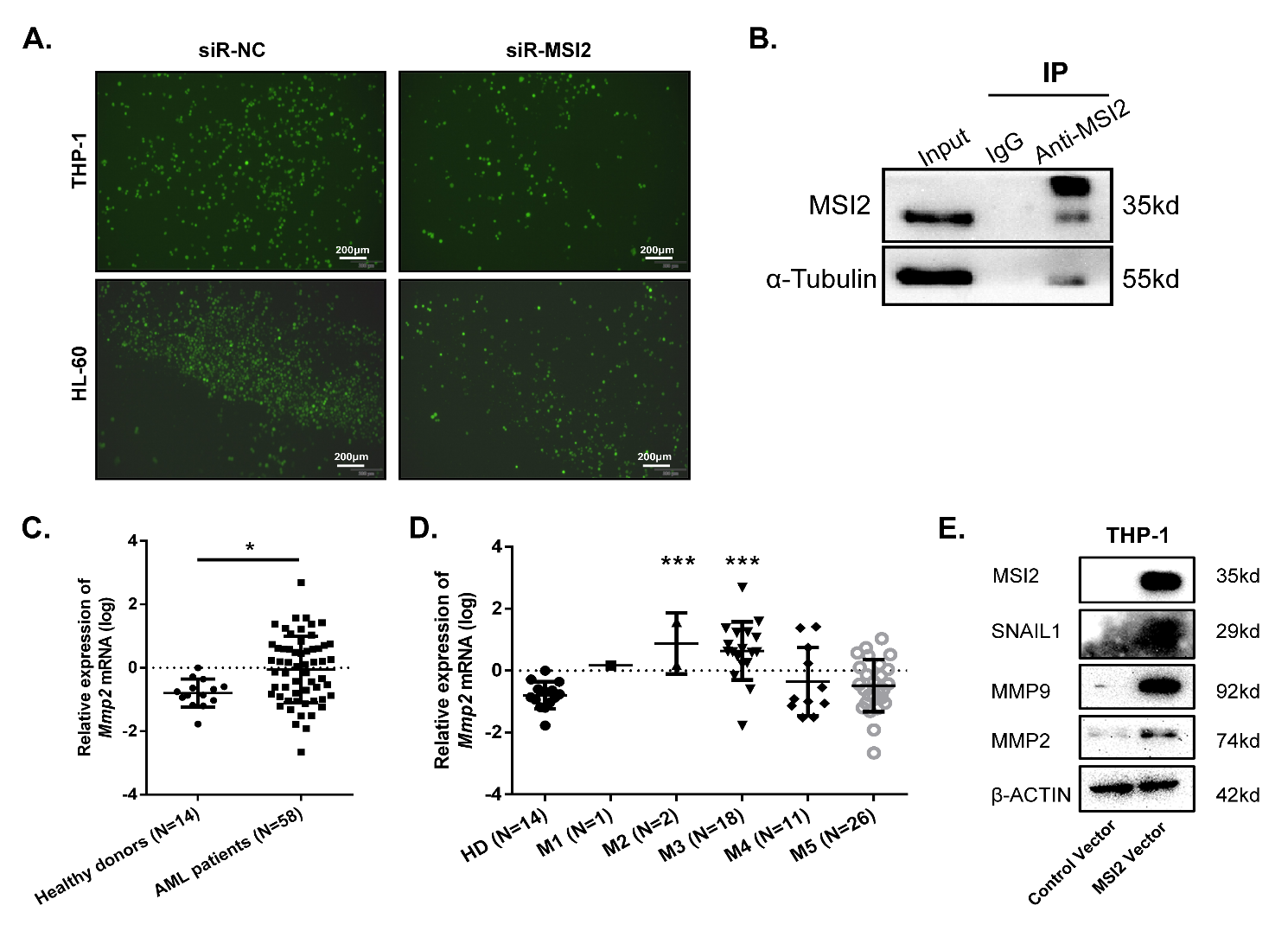


**Figure S6. Migration effect of MSI2 in AML**.

**(A)** Cell migration in EGFP-labeled THP-1 and HL-60 cells transfected with siR-MSI2 or siR-NC was detected by transwell assay. Representative pictures from three independent experiments were observed by fluorescence microscope, scale bar = 200 μm. **(B)** Western blotting was used to detect MSI2 protein after RIP-PCR. **(C)** Relative expression of *Mmp2* mRNA in healthy donors (*N*=14) and primary AML patients (*N*=58) was measured by qRT-PCR. **(D)** Comparison of the relative expression of *Mmp2* mRNA between different AML types. **(E)** Expression of MSI2 and tumor metastasis-associated proteins in Lenti- MSI2 vector- or Lenti- Control vector-transfecting THP-1 cells. Data are expressed as mean ± SD (error bars). * *P* < 0.05, ****P* < 0.001, *t*-test.

**Figure S7**

**
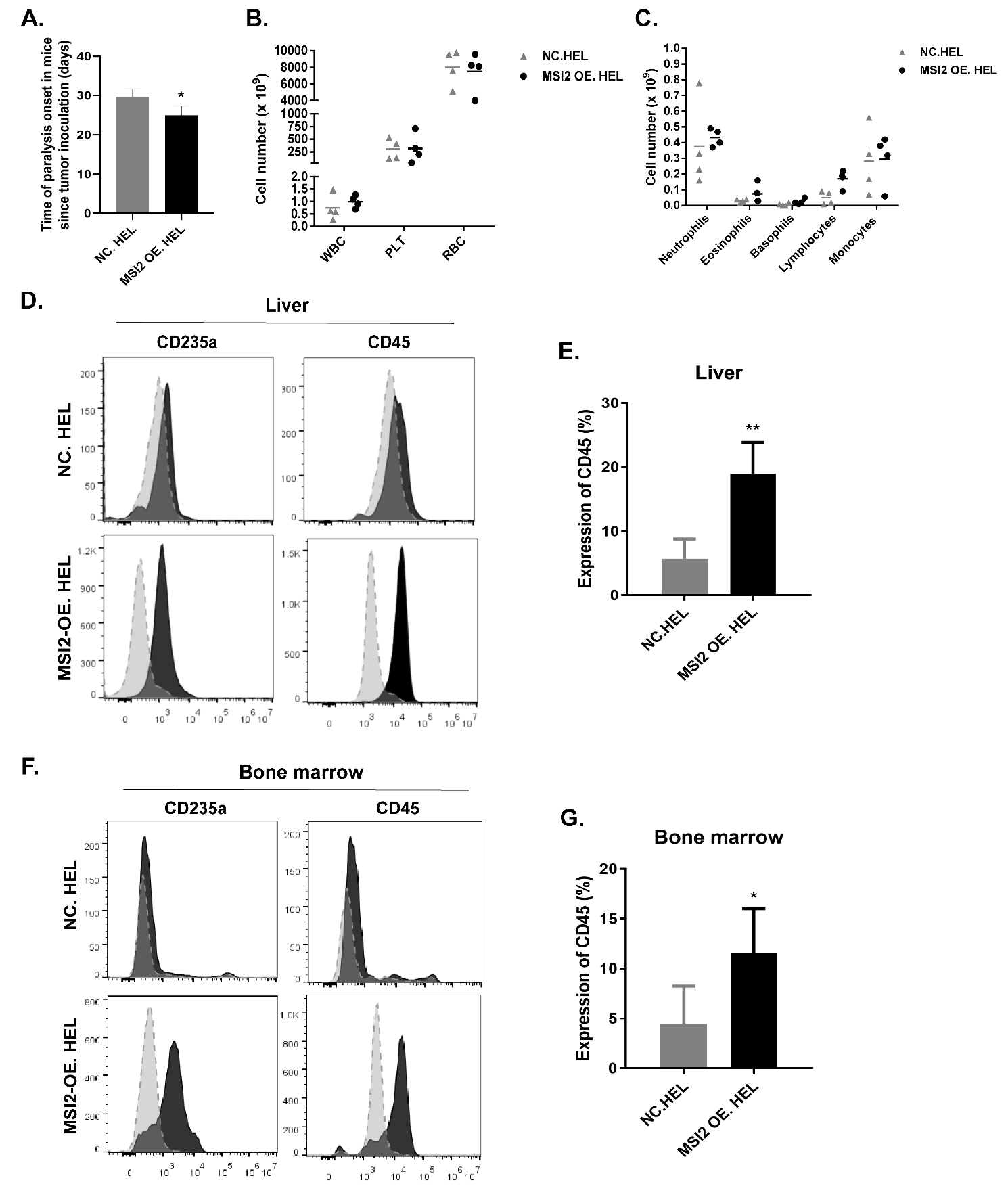
**

**Figure S7. Flow cytometric analysis for human antigen expression in mouse tissues**.

**(A)** NOG mice were intravenously inoculated with 5.0×10^6^ NC.HEL or MSI2-OE. HEL cells. Time of paralysis onset in mice since tumor inoculation between NC. HEL group and MSI2-OE. HEL group. **(B)** White blood cell (WBC), platelet (PLT) and red blood cell (RBC) from peripheral blood in two groups of mice were counted by complete blood count after mice were euthanized. Horizontal lines denote mean values, *N*=4. **(C)** Neutrophils, eosinophils, basophils, lymphocytes and monocytes from peripheral blood in two groups of mice were counted. Horizontal lines denote mean values, *N*=4. **(D, F)** Representative flow graph of CD235a and CD45 expression in liver (D) and bone marrow (F). The gray dashed line represents isotype control; the black solid line represents experimental group stained with fluorescent antibodies. **(E, G)** Statistical analysis of CD45 expression in liver (E) and bone marrow (G) from each group of mice. Data are expressed as mean ± SD (error bars). * *P* < 0.05, ** *P* < 0.01, *t*-test.

**Figure S8**


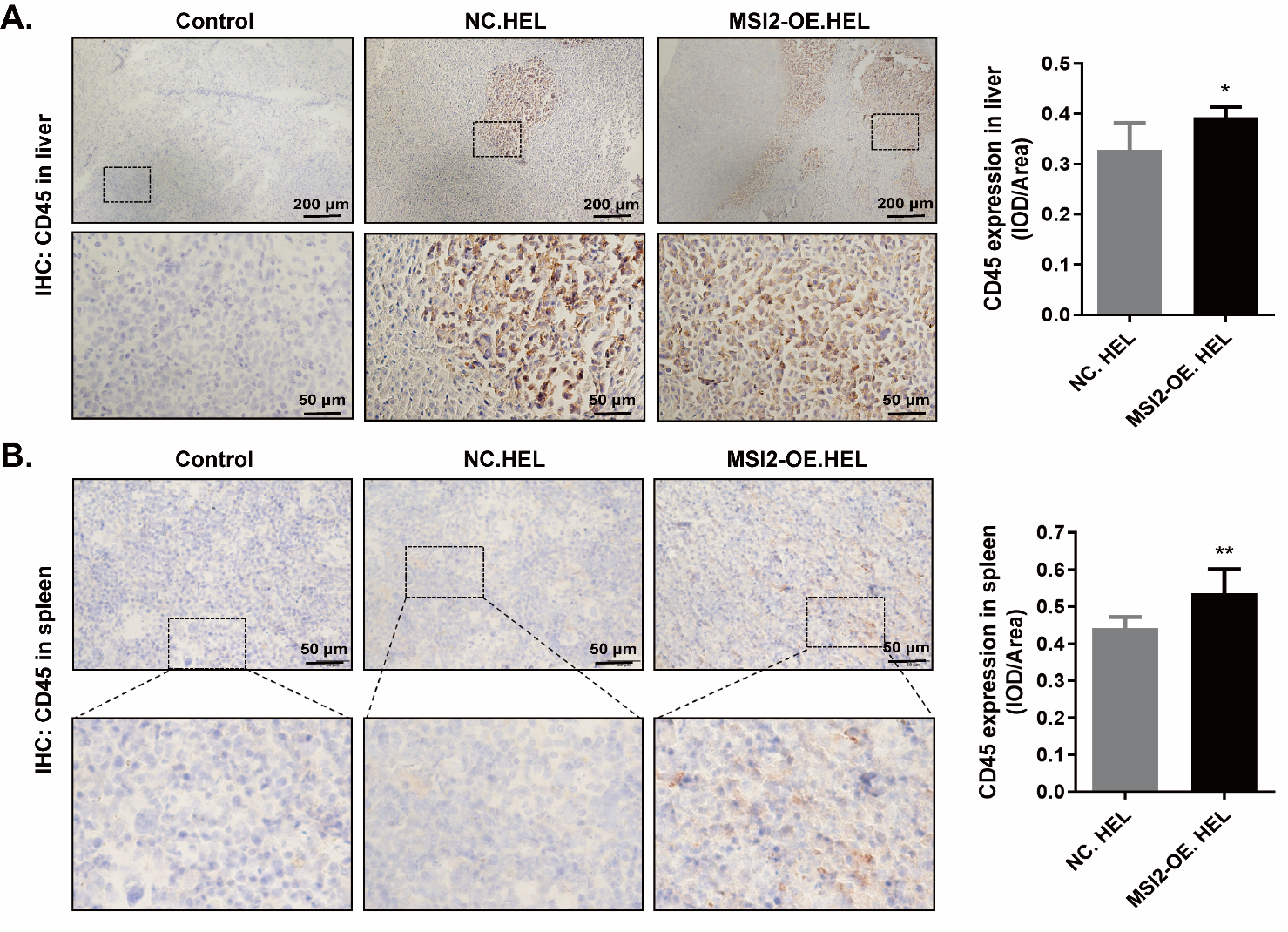


**Figure S8. IHC analysis for human antigen expression in mouse tissues**.

**(A, B)** CD45 expression in liver (A) and spleen (B) from each group of mice was detected by IHC. Representative pictures (left panel) and statistical analysis diagram (right panel) were illustrated. Data are expressed as mean ± SD (error bars). * *P* < 0.05, ** *P* < 0.01, *t*-test.

**Figure S9**

**
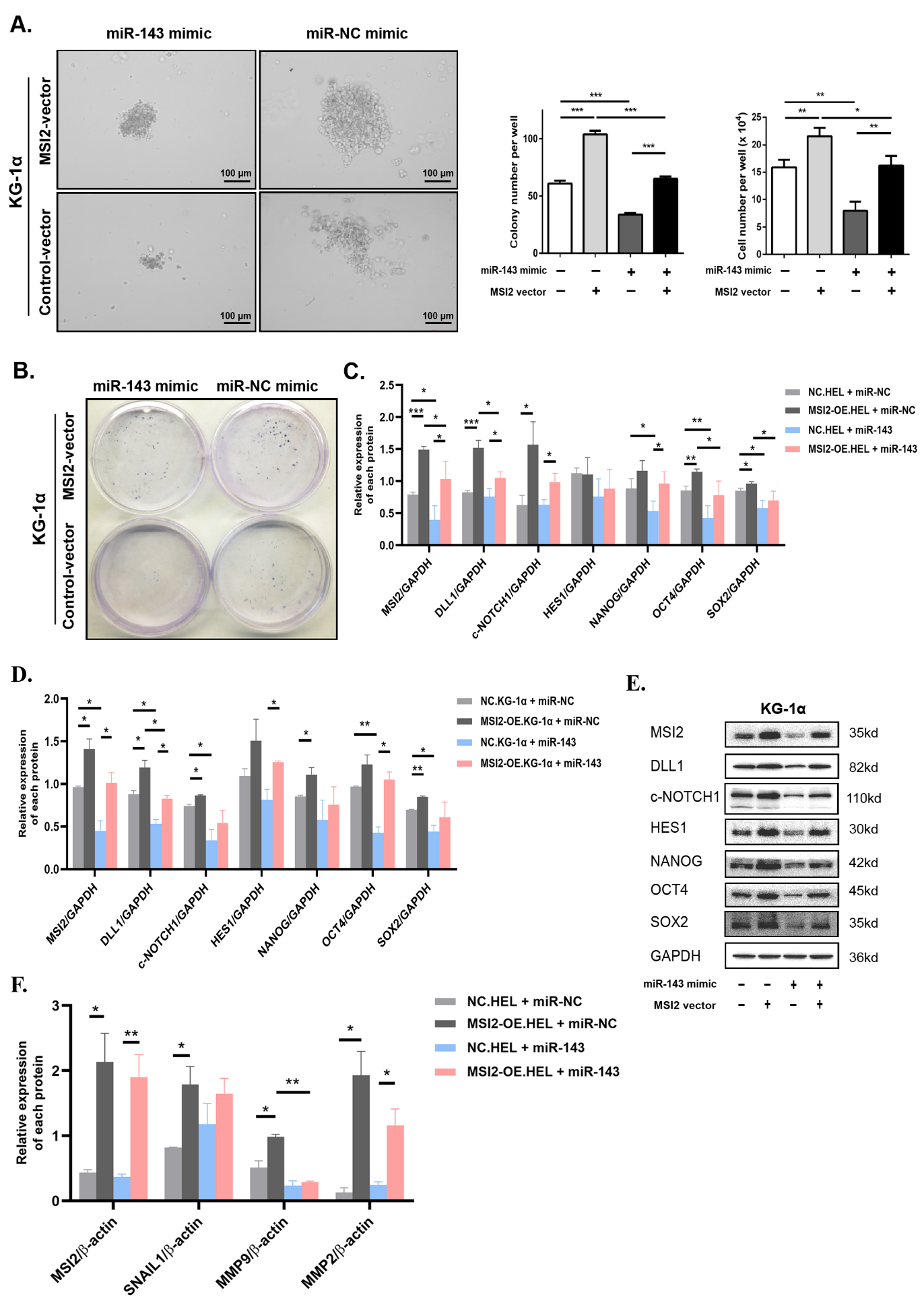
**

**Figure S9. MSI2 activity was negatively modulated by miR-143 in AML cells**.

**(A)** Colony-forming capacity of Lenti- MSI2 vector- or Lenti- Control vector-transfecting KG-1α cells after the transfection with miR-143 or miR-NC mimic. Representative pictures (Above) and statistical analysis diagram (Below) were illustrated, scale bar = 200 μm. **(B)** Colonies in (A) were stained with crystal violet. **(C)** Statistical analysis of relative expression of MSI2, key factors of Notch1 signaling pathway and cancer stemness-related proteins in Lenti- MSI2 vector- or Lenti- Control vector-transfecting HEL cells after the transfection with miR-143 or miR-NC. **(D, E)** Relative expression of MSI2, key factors of Notch1 signaling pathway and cancer stemness-related proteins in Lenti- MSI2 vector- or Lenti- Control vector-transfecting KG-1α cells after the transfection with miR-143 or miR-NC. Representative pictures (E) and statistical analysis diagram (D) were illustrated. **(F)** Statistical analysis of relative expression of MSI2 and tumor metastasis-associated proteins in Lenti- MSI2 vector- or Lenti- Control vector-transfecting HEL cells after the transfection with miR-143 or miR-NC. Data are expressed as mean ± SD (error bars). * *P* < 0.05, ** *P* < 0.01 and ****P* < 0.001, *t*-test.

**Figure S10**


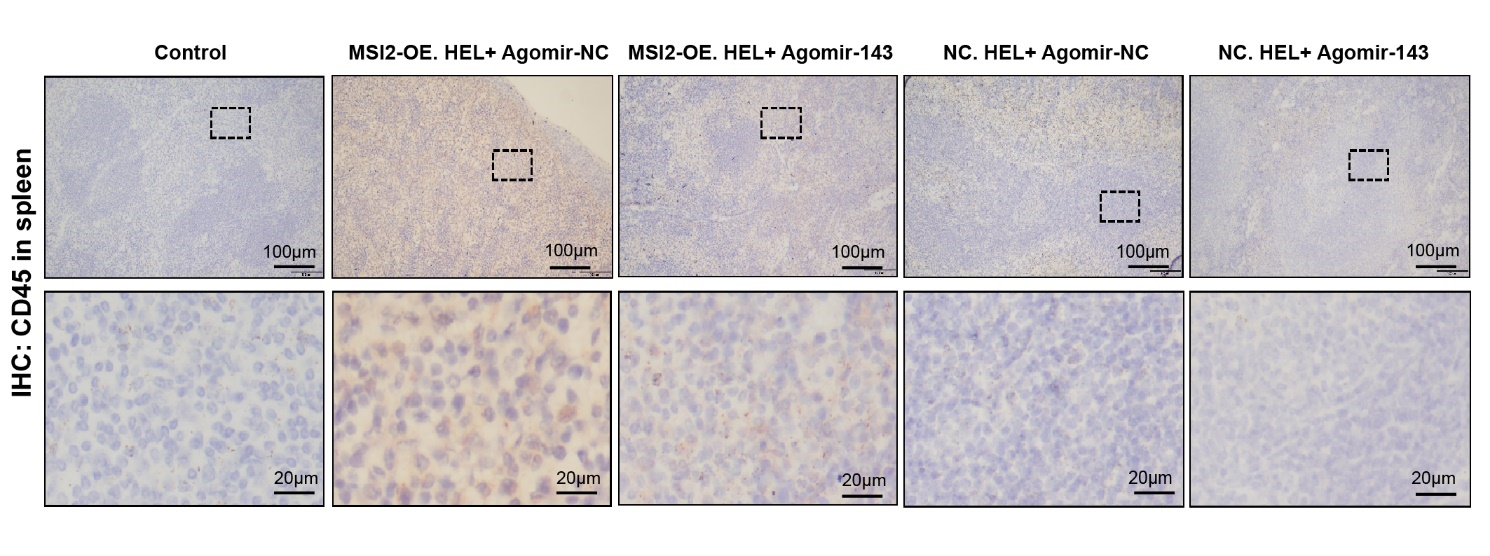


**Figure S10. Expression of CD45 in spleen was detected by IHC.**

BALB/c-Nu mice were incubated with 5×10^6^ MSI2-OE. HEL cells or NC. HEL cells subcutaneously to establish xenograft AML model, and then treated intratumorally with micrOFFTM agomir-143 or agomir-NC every three days for two weeks. After mice were euthanized, spleen tissues were cut into sections and stained with anti-human CD45 antibody for analyzing the metastatic ability of AML xenograft.

**Figure S11**


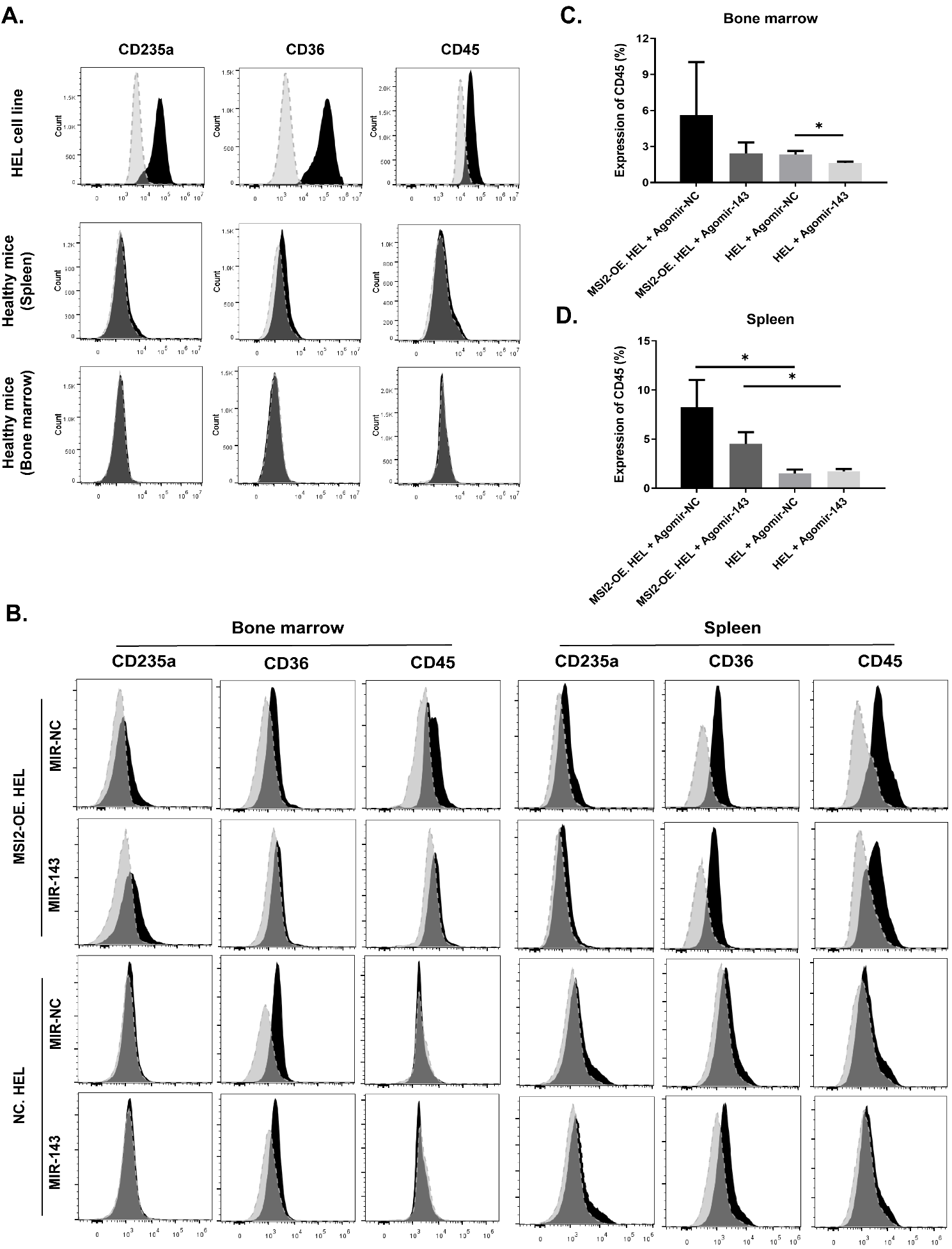


**Figure S11. Flow cytometric analysis for human antigen expression**. **(A)** Representative flow graph of human CD45, CD36 and CD235a expression. HEL cell line was served as a positive control; spleen and bone marrow samples from healthy mice were served as a negative control, *N*=4. **(B)** Representative flow graph of human CD45, CD36 and CD235a expression in bone marrow (left panel) and spleen (right panel), *N*=4. The gray dashed line represents isotype control; the black solid line represents experimental group stained with fluorescent antibodies. **(C, D)** Statistical analysis of CD45 expression in bone marrow (C) and spleen (D) of each group of mice, *N*=4. Data are expressed as mean ± SD (error bars). * *P* < 0.05, ** *P* < 0.01 and ****P* < 0.001, *t*-test.

**Figure S12**

**
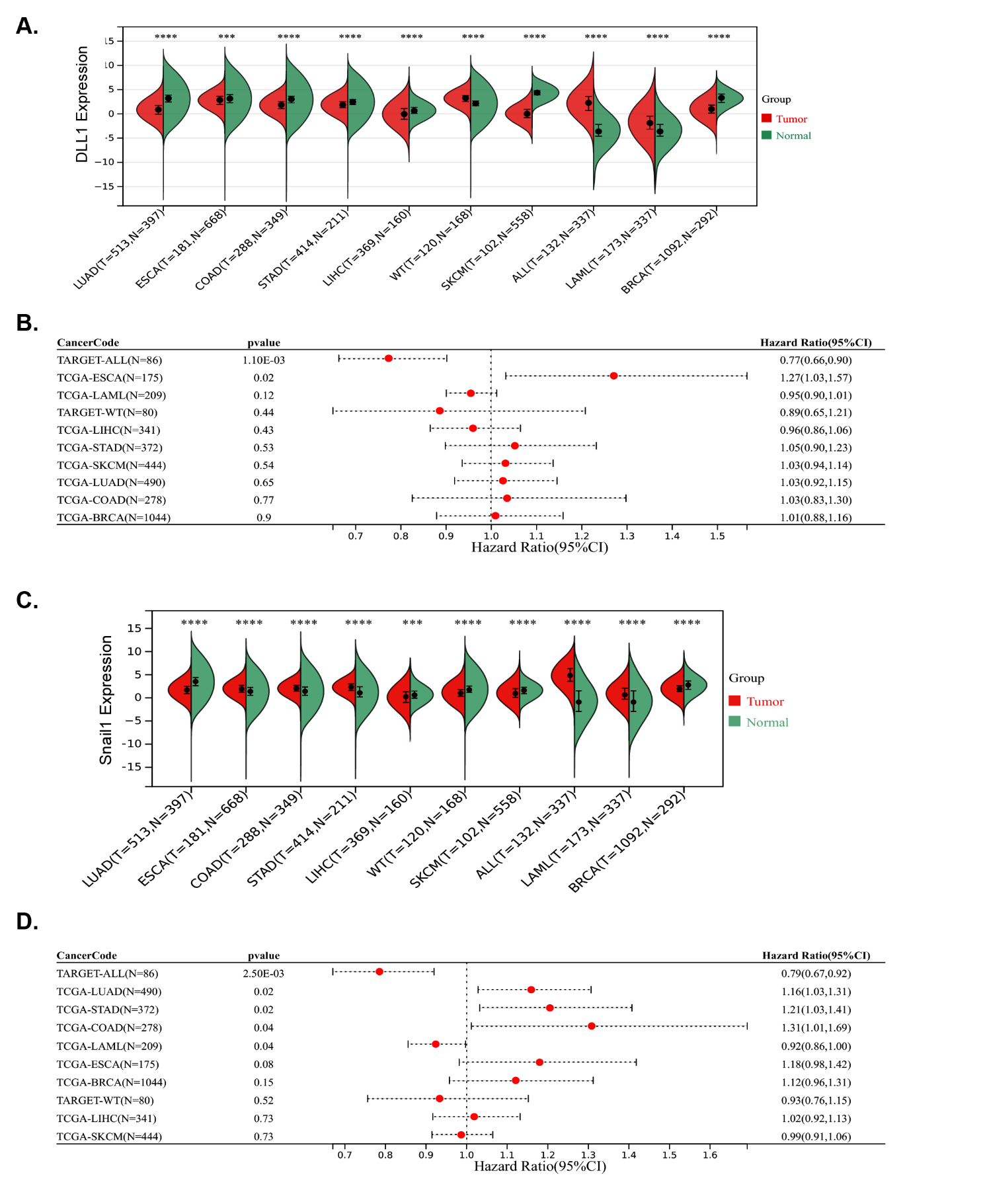
**

**Figure S12. *DLL1* and *Snail1* gene expression in clinic**. **(A, C)** *DLL1* (A) and *Snail1* (C) gene expression between normal and tumor samples in log2(x+0.001) transform from the UCSC database (TCGA, TARGET, GTEx) was analyzed, Unpaired Wilcoxon Rank Sum and Signed Rank Tests. **(B, D)** Relationship between the expression and prognosis of DLL1 (B) and Snail1 (D) in each tumor was analyzed by Cox proportional hazards regression model established by coxph function of the R package survival, Log-rank test. LUAD: Lung adenocarcinoma; ESCA: Esophageal carcinoma; COAD: Colon adenocarcinoma; STAD: Stomach adenocarcinoma; LIHC: Liver hepatocellular carcinoma; WT: High-Risk Wilms Tumor; SKCM: Skin Cutaneous Melanoma; ALL: Acute Lymphoblastic Leukemia; LAML: Acute Myeloid Leukemia; BRCA: Breast invasive carcinoma.
